# New Challenges in Psycho‐Oncology Research III: A systematic review of psychological interventions for prostate cancer survivors and their partners: clinical and research implications

**DOI:** 10.1002/pon.4431

**Published:** 2017-07-10

**Authors:** Suzanne K. Chambers, Melissa K. Hyde, David P. Smith, Suzanne Hughes, Susan Yuill, Sam Egger, Dianne L. O'Connell, Kevin Stein, Mark Frydenberg, Gary Wittert, Jeff Dunn

**Affiliations:** ^1^ Menzies Health Institute Queensland Griffith University Gold Coast Queensland Australia; ^2^ Cancer Research Centre Cancer Council Queensland Brisbane Queensland Australia; ^3^ Prostate Cancer Foundation of Australia Sydney New South Wales Australia; ^4^ Health & Wellness Institute Edith Cowan University Perth Australia; ^5^ Institute for Resilient Regions University of Southern Queensland Toowoomba Queensland Australia; ^6^ Australian and New Zealand Urogenital and Prostate Cancer Trials Group Sydney New South Wales Australia; ^7^ Cancer Research Division Cancer Council NSW Sydney New South Wales Australia; ^8^ Sydney Medical School‐Public Health University of Sydney Sydney New South Wales Australia; ^9^ School of Medicine and Public Health University of Newcastle Newcastle New South Wales Australia; ^10^ Behavioral Sciences and Health Education, Rollins School of Public Health Emory University Atlanta Georgia USA; ^11^ Department of Urology Monash Health Melbourne Victoria Australia; ^12^ Department of Surgery, Faculty of Medicine Monash University Melbourne Victoria Australia; ^13^ Freemasons Foundation Centre for Men's Health, School of Medicine University of Adelaide Adelaide South Australia Australia

## 1 BACKGROUND

The medical and social context of prostate cancer (PCa) has changed dramatically since the introduction of PSA testing for early detection in the late 1980s,^1^ leading to a peak in incidence in the developed world in the 1990s and again a decade later.^2^ Since that time, novel PCa treatments have rapidly emerged in the radiation and medical oncology field, as well as surgical advances.^3^ The recent emergence of active surveillance for low‐risk disease has further expanded possible treatment approaches.^4^ Market forces from consumers, clinicians, and the therapeutic industry have driven changes in clinical and surgical management and treatment; however, psycho‐oncological research and survivorship care arguably has lagged behind. Specifically, although men are surviving longer, they may not be surviving well. In 2012, there were over 1.1 million incident cases of PCa diagnosed and more than 300 000 deaths worldwide.^5^ Five‐year prevalence estimates suggest that there are over 3.8 million PCa survivors globally^6^ with this expected to increase rapidly in future.^7^ The challenges we face in meeting the needs of these men and their families into the future are vast.

Up to 75% of men treated for localised PCa report severe and persistent treatment side‐effects including sexual dysfunction, poor urinary or bowel function.^8^ Psychosocial concerns are prevalent with 30%‐50% of PCa survivors reporting unmet sexuality, psychological, and health system and information needs^9^, ^10^ and 10%‐23% of men clinically distressed.^11^ Risk of suicide is increased after PCa diagnosis^12^, ^13^ and can persist for a decade or more.^14^ In the longer term, 30%‐40% of PCa survivors report persistent health‐related distress, worry, low mood^15^ and diminished quality of life (QoL).^16^ Partners of PCa survivors also experience ongoing psychological concerns and changes in their intimate relationships^17^; with these impacts driven in part by the man's level of distress, sexual concerns and physical QoL.^18^

In 2011, our group published the first criterion‐based systematic review of psychosocial interventions for men with PCa and their partners.^19^ We concluded that group cognitive‐behavioural interventions and psycho‐education appeared to be helpful in promoting better psychological adjustment and QoL for men with localised PCa, and coping skills training for female partners may improve their QoL. However, data were limited by inconsistent results and low study quality. In response to the increasing burden of PCa, uncertainties about optimal psychosocial care, and additions to the literature, we updated and extended this review with the intent of determining benefit and acceptability, and considering intervention content and format. In brief, we considered the range of psychosocial and psychosexual interventions that may be optimal, and for whom.

## 2 METHODS

Two clinical questions guided the review^20^: In men diagnosed with PCa (Q1) and/or in their partners/carers (Q2), what is the effectiveness of different psychosocial or psychosexual interventions compared with (i) other psychosocial or psychosexual interventions, or (ii) usual care or no intervention, in maintaining or improving QoL or psychological wellbeing? Psychosocial or psychosexual interventions were included if they had one or more of the following components: education (psycho‐education, psycho‐sexual education, PCa education), cognitive‐behavioural (cognitive restructuring, behaviour change, cognitive‐behavioural stress management), relaxation (relaxation techniques, meditation), supportive counselling (counselling/psychotherapy, health professional discussion), peer support (peer support, social support including discussion within a group of peers), communication (skill development to encourage communication with partners, health professionals or generally) and decision support (aids or tools to assist decisions about PCa treatment or use of sexual aids). The review and reporting of results were guided by the PRISMA statement.^21^ Ethical approval was not required.

### 2.1 Search strategy

**Figure 1:**
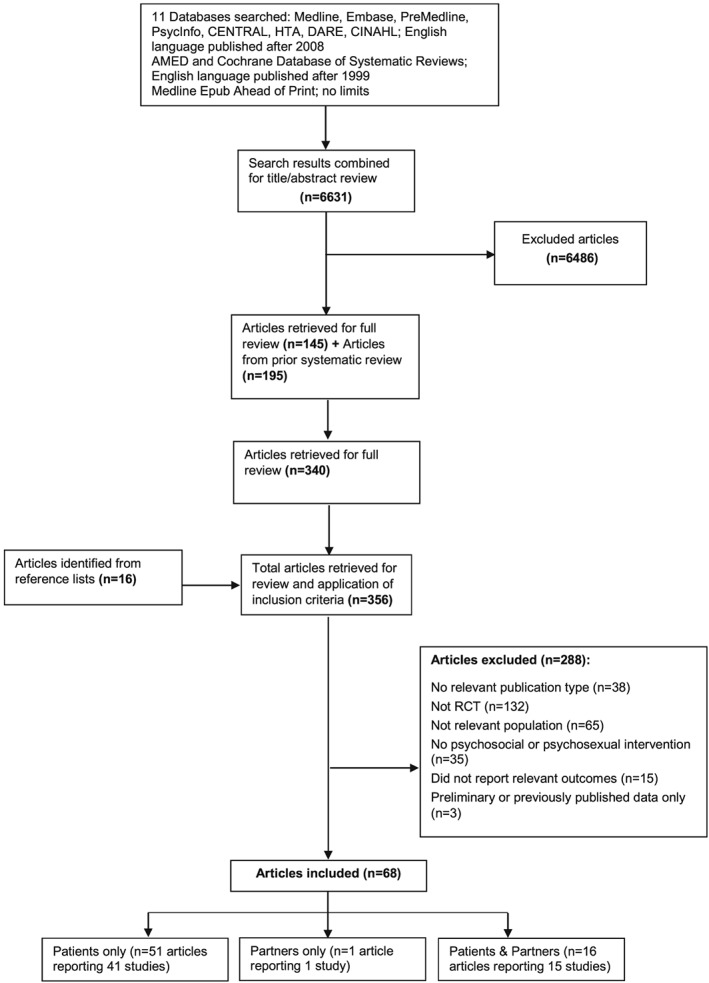
PRISMA flow diagram of study selection for systematic review

Our prior review (until December 1, 2009) identified 195 articles that met criteria for the current study.^19^ Searches were updated from 2009 onwards. Eleven relevant databases were searched (eg, MEDLINE, Embase, PsycINFO, and CINAHL; Figure 1) up to January 9, 2017. Free‐text terms and database‐specific subject headings for PCa and psychological and QoL outcomes were used (Appendix A shows full search strategies). Reference lists of included articles were also searched. ClinicalTrials.gov (http://clinicaltrials.gov/) (June 2016) and the International Clinical Trials Registry Platform (http://apps.who.int/trialsearch/) (October 2016) were searched for ongoing and completed trials and associated publications.

### 2.2 Selection criteria

Studies were included if the following pre‐specified criteria were met:

- Randomised controlled trial design.
- ≥80% of participants were men diagnosed with PCa (no restrictions on disease stage or time since diagnosis) and/or partners/carers of men with PCa or results for men with PCa and/or partners/carers were reported separately.
- Intervention(s) were psychosocial or psychosexual.
- Outcome(s) reported were psychosocial (including psychological, relationships, decision‐making), health‐related QoL, and sexuality outcomes (including sexual function, bother, and use of erectile dysfunction aids or treatments). Mediator outcomes such as cognitive reframing and coping were not included.
- Outcomes were assessed using validated scales or scales adapted from these.
- Intervention(s) were compared with usual care or supportive attention or no intervention, and/or another intervention(s) with different psychosocial or psychosexual components, and/or the same intervention components with a different mode(s) of delivery. Multimodal interventions such as lifestyle interventions were only included if they had a psychosocial or psychosexual component.
- Published in English language.
- Published after December 31, 1999 up to January 9, 2017.

Two authors reviewed titles and abstracts and excluded irrelevant articles and duplicates. Full‐text articles that potentially met criteria were then retrieved and reviewed by one author. A random sample of 5% of articles was assessed for inclusion by 2 authors with 100% agreement achieved.

### 2.3 Data extraction

One author extracted pre‐specified study characteristics (eg, participant demographics, PCa treatments, intervention content, delivery and results) and another checked each extract. To support data extraction, published descriptions of interventions were content analysed to create a framework of common psychosocial or psychosexual intervention components (Appendix B).

### 2.4 Risk of bias

The Cochrane Collaboration's tool was used to assess risk of bias regarding sequence generation, allocation concealment, blinding of participants and personnel collecting outcome data, incomplete outcome data, selective outcome reporting, and other sources (eg, difference in follow‐up between arms).^22^ Blinding is difficult to achieve in psychological trials where consent mechanisms require participants to understand differences in treatments, which are often clearly discernible to the participant (eg, therapist‐delivered intervention vs self‐help materials).^19^ On this basis, blinding was excluded from assessment. Clinical trial registries at https://clinicaltrials.gov/, http://www.isrctn.com/, and http://www.anzctr.org.au/ were searched for protocols of included studies to identify pre‐specified outcomes and determine whether there was a risk of bias from selective outcome reporting. Differences in evaluations were resolved by discussion and where necessary adjudication by a third author.

### 2.5 Intervention acceptability

The criteria of Yanez et al^23^ were used to identify and evaluate aspects of interventions that indicate acceptability: ≥40% recruitment rate, ≥70% retention at end of intervention or follow‐up (or <30% withdrawal), and ≥70% average intervention attendance.

### 2.6 Analyses

It was anticipated that some trials may be underpowered.^19^ Thus, an intervention was considered potentially beneficial compared with usual care or better than another intervention if for at least one reported outcome (at the longest reported follow‐up), there was in favour of the intervention(s): (i) a statistically significant difference between arms; (ii) a moderate or large standardised effect size (eg, Cohen's *d* ≥ 0.5, *η* ^2^ ≥ 0.06); or (iii) a difference in mean score changes from baseline calculated by ANCOVA or multiple linear regression between arms ≥10% of the scale of the differences in means. For a given measurement scale, results from subscales were only considered in the absence of an overall score.

## 3 RESULTS

### 3.1 Search results

In all, 6631 citations were identified of which 161 full‐text (including 16 identified from reference lists) were retrieved and evaluated as well as 195 articles from the prior review.^19^ Of the total 356 full‐text articles assessed for inclusion, 68 articles met criteria and reported a total of 57 RCTs. Forty‐one RCTs reported in 51 articles (2 publications for 10 studies) included only patients (Q1); 1 RCT included only partners (Q2); 15 RCTs reported in 16 articles (2 publications for 1 study) included patients and partners (Q1 and Q2) (Figure 1). Most studies were excluded because of study design or population not meeting criteria, or results for patients or partners/carers were not reported. Clinical trial registry searches identified 47 trials: 25 completed (16 included in the review); 20 ongoing; 2 terminated (slow accrual, funding unavailable).

### 3.2 Risk of bias

Risk of bias from sequence generation (61% Q1; 64% Q2) and allocation concealment (71% Q1; 79% Q2), was unclear, and high for incomplete outcome data (43% Q1; 43% Q2) for most studies. Risk of bias from selective outcome reporting was also high for majority of partner studies (43%) and unclear for patient studies (63%). Most studies were low risk for other sources of bias (70% Q1; 86% Q2) (Appendix C).

### 3.3 Trial characteristics

Included trials randomised 8378 men (range 27‐740; 48% of trials had <100 participants), and 1313 partners (range 27‐263; 57% of trials had <100 participants; >90% partners were female in 14 trials; >80% partners were spouses in 12 trials). Most (67%) trials were conducted in North America. In 10 trials (4 including partners), participation was determined by socio‐demographic background (eg, African‐American), emotional state (eg, distress), or QoL (eg, urinary or sexual dysfunction, ADT treatment side‐effects, fatigue). When reported, mean or median age was below 65 years in 49% of trials for patients and below 65 years in 100% of trials for partners. In approximately half of trials (57% of patient trials, 40% of partner trials) reporting college/university education, >50% of participants were university/college educated. In 25 trials (45%), men were diagnosed with or treated for localised disease in the previous 6 months (14 trials enrolled men prior to treatment or treatment decision). Men with recurrent or metastatic disease and their partners were included in 16% and 21% of trials, respectively.

The number of relevant outcomes measured by trials varied from 1 to 16 (patient) and 2 to 12 (partner). Most common outcomes for patients were sexual bother and/or function and mental health; and for partners were relationships, general and cancer‐specific distress. Trials reported 41 patient, 1 patient and partner, and 1 partner person‐focused (targeted and delivered to the individual or person) interventions and 14 couple‐focused interventions (targeted and delivered to the couple as a dyad) (Appendix D). Most interventions were compared with usual or standard care; however, what the comparison group entailed was rarely described. Follow‐up ranged from immediately post‐intervention to approximately 19 months (person‐focused, Median = 3 months) or 12 months (couple‐focused, Median = 6 months) post‐intervention.

### 3.4 Intervention acceptability

Trials comprising interventions that were person‐focused were more acceptable than couple‐focused interventions (recruitment: 72% vs 29%; retention: 74% vs 64%). Approximately 40% of person and couple interventions indicated acceptable mean attendance (Table 1).

**Table 1:** Acceptability of included trials comprising person‐ (*n* = 43) and couple‐ (*n* = 14) focused interventions

Acceptability category	Person* *N* (%)	Couple *N* (%)
1. Recruitment		
No: <40%	8 (19%)	6 (43%)
Yes: ≥40%	31 (72%)	4 (29%)
Unclear: Not reported	4 (9%)	4 (29%)
2. Retention/Withdrawal		
No: Retention <70%; Withdrawal > 30%	2 (5%)	1 (7%)
Yes: Retention ≥70%; Withdrawal ≤ 30%	32 (74%)	9 (64%)
Unclear: Not reported	9 (21%)	4 (29%)
3. Attendance		
No: <70%	7 (16%)	2 (14%)
Yes: ≥70%	18 (42%)	6 (43%)
Unclear: Not reported	18 (42%)	6 (43%)

* Includes 2 person‐focused trials for partners both rated acceptable on recruitment, retention, and attendance.

### 3.5 Intervention effects

Three trials reported couple‐focused interventions that, compared with usual care, increased partner distress about sexual function,^24^ worsened partner challenge appraisal,^25^ and reduced relationship satisfaction and intimacy for partners who had high levels of these constructs at baseline^26^ (Appendix D). By contrast, for patients, all intervention effects indicated improvement. Four trials included outcomes of interest^27^, ^28^, ^29^, ^30^ but did not report comparative results and were excluded. The remaining 29 trials (21 person‐focused: 20 patients, 1 partner and patient; 8 couple‐focused) showed a benefit for psychosocial or psychosexual outcomes (Table 2). Most (80%) person‐focused interventions were for men with localised disease. Of the effective interventions, most (95% person‐focused, 86% couple‐focused) significantly impacted patient outcomes. No person‐focused trials had a significant effect on relationship outcomes. No couple‐focused trials improved decision‐making outcomes or fatigue. No trials had a significant effect on partner QoL or sexuality outcomes regardless of intervention focus. Table 3 reports intervention components.

**Table 2:** Person ‐ (*N* = 21) and couple ‐ (*N* = 8) focused trials that significantly (or moderate‐large effect size) and positively impacted psychosocial or psychosexual outcomes

Study	N	Intervention(s) that had an effect	Comparison	Components	Deliverer	Follow‐up	Outcomes impacted	Sig level or effect size *
Person‐focused interventions								
Badger 2011,2013 *Patients + partners*	71	1. Interpersonal psychotherapy + cancer education: patient and partner	2. Health education attention: patient and partner	1. E, SC, PS, C 2. E	1. Nurse or social worker 2. Research assistants	8 weeks post‐intervention	Depression • Patient • Partner Negative affect • Patient Stress • Patient Fatigue • Patient • Partner Social well‐being • Partner Spiritual well‐being • Patient • Partner	*P* < 0.001 *P* < 0.05 *P* < 0.001 *P* < 0.001 *P* < 0.01 *P* < 0.01 *P* < 0.01 *P* < 0.01 *P* < 0.01
		8 (patients) or 4 (partners) individual telephone sessions over 8 weeks						
Bailey 2004	39	Uncertainty management: cognitive reframing tailored to patient needs 5 weekly individual telephone sessions	UC	E, CB, C, DS	Nurse	~5 weeks post‐intervention	QoL	*P* = 0.01
Berry 2012,2013	494	Decision support 1 individual internet session	UC	E, C, DS	Self‐admin	6 months post‐intervention	Decisional uncertainty	*P* = 0.04
Campo 2014	40	Qigong 24 twice weekly group face‐to‐face sessions	Stretch control	R	Qigong master and instructors	1 week post‐intervention	Fatigue Distress	*P* = 0.02 *P* = 0.002
Carmack‐Taylor 2006,2007	134	1. 30 minutes expert speaker or facilitated discussion 2. 90 minutes expert speaker or facilitated discussion Both interventions 21 group face‐to‐face sessions over 6 months	UC	1. E, PS 2. E, PS	Facilitator supervised by clinical psychologist	6 months post‐intervention	Anxiety Depression	Sub‐group *P* = 0.02 Sub‐group *P* = 0.002
Chabrera 2015	142	Decision aid Individual printed	UC	E, C, DS	Self‐admin	3 months post‐baseline	Decisional conflict	*P* < 0.001
Chambers 2013	740	Telephone psycho‐educational 5 individual sessions: 2 pre‐tx, and 3 weeks, 7 weeks and 5 months post‐tx	UC	E, CB, R, DS	Nurse Counsellor	24 months post‐tx	Cancer‐specific distress Mental health	Sub‐group *P* < 0.008 Sub‐group *P* = 0.04
Diefenbach 2012	91	1. Prostate Interactive Educational System with or without tailoring to patient's information seeking style *(combined results from arms)* 1 individual internet/CD‐ROM session	2. Control Read Standard National Cancer Institute booklets on PCa for 45 minutes 1 individual booklet	1. E, DS 2. E	Self‐admin	Immediately post‐intervention	Confident about tx choice Prefer more information	*P* = 0.02 *P* = 0.02
Hacking 2013	123	Decision navigation 1 individual face‐to–face or telephone session, audiotape and written notes	UC	DS	Research assistants	6 months post‐consult	Decisional self‐efficacy Decisional regret	*P* = 0.009 *P* = 0.04
Lepore 2003; Helgeson 2006	250	1. Education + group discussion (with family member/friend) 2. Education Both 6 weekly face‐to‐face group sessions	Standard medical care	1. E, PS 2. E	Multiple health professionals	12 months post‐ intervention	Mental health Depression Sexual bother	Sub‐group *P* < 0.05 Sub‐group *P* < 0.05 *P* < 0.01
Mishel 2009	252	1. Decision navigation: Patient only 2. Decision navigation: Patient and support person Both information + telephone calls to review content, identify/formulate questions and practise skills delivered to patient and/or support person individually (not dyad) Both individual/couple booklet, DVD and 4 telephone calls over 7‐10 days	Control	1. E, SC, C, DS 2. E, SC, C, DS	Nurse, Self‐admin	3 months post‐baseline	Decisional regret	*P* = 0.01
Penedo 2006; Molton 2008	191	1. 10‐week group CB stress management techniques + relaxation training 10 weekly group face‐to‐face sessions	2. Half‐day stress management seminar (same content) 1 group face‐to‐face session	1. E, CB, R, SC, PS, C 2. E	Therapist	12‐13 weeks post‐baseline	Cancer‐related QoL Sexual function	*P* < 0.05 Sub‐group *P* < 0.05
Penedo 2007	93	1. 10‐week group CB stress management techniques + relaxation training 10 weekly group face‐to‐face sessions	2. Half‐day stress management seminar (same content) 1 group face‐to‐face session	1. E, CB, R, SC, PS, C 2. E	Therapist	12‐13 weeks post‐baseline	Cancer‐related QoL	*P* = 0.006
Petersson 2002	118	Group rehabilitation programme (only or + individual support) including psychosocial components + physical activity 8 group face‐to‐face sessions over 8 weeks + booster group session after 2 months + written information	No group intervention	E, CB, R	Multiple health professionals	3 months post‐intervention start	Cancer‐related distress (Avoidance)	Sub‐group *P* < 0.01
Schofield 2016	331	Nurse‐led group psycho‐educational consultation 4 x group face‐to‐face sessions (beginning, mid, completion, and 6 weeks post‐radiotherapy) + 1 individual session after 1st group consultation	UC	E, PS, C	Uro‐oncology nurse	6 months post‐tx	Depression	*P* = 0.0009
Siddons 2013	60	CB group intervention 8 group face‐to‐face sessions over 8 weeks	Wait‐list	E, CB, R, C	Psychologist	8 weeks (end of intervention)	Masculine self‐esteem Sexual confidence Sexual QoL Orgasm satisfaction	*P* = 0.037 *P* = 0.001 *P* = 0.046 *P* = 0.047
Traeger 2013	257	1. 10‐week group CB stress management techniques + relaxation training 10 weekly group face‐to‐face sessions	2. Half‐day stress management seminar (same content) 1 group face‐to‐face session	1. E, CB, R, SC, PS, C 2. E	Therapist	12‐13 weeks post‐baseline	Emotional well‐being	*P* < 0.05
Weber 2004	30	Peer support 8 individual face‐to‐face sessions over 8 weeks	UC	PS	Peer (>3 years PCa survivor)	8 weeks post‐baseline	Sexual bother	*P* = 0.014
Weber 2007 a,b	72	Peer support 8 individual face‐to‐face sessions over 8 weeks	UC	PS	Peer (>3 years PCa survivor)	8 weeks post‐baseline	Depression Self‐efficacy	*P* = 0.03 *P* = 0.005
Wootten 2015, 2016	142	1. Online psycho‐education + moderated peer online forum (*PsychE + F*) 6 individual sessions over 10 weeks	2. Moderated peer online forum (*F*) Individually accessed over 10 weeks	1. E, CB, PS, C 2. PS	Self‐admin	6 months post‐baseline	Distress Decisional regret Sexual satisfaction	*P* = 0.02 *P* = 0.046 Sig level NR, Difference 1.24 (95%CI 0.25‐2.22)
Yanez 2015	74	1. CB stress management + relaxation/stress reduction techniques 10 weekly group online sessions	2. Health promotion attention‐control 10 weekly group online sessions	1. E, CB, R, PS, C 2. E	Therapist	6 months post‐baseline	Depression	Cohen's d 0.5
Couple‐focused interventions								
Campbell 2007	30	Partner assisted coping skills training 6 ~weekly dyadic telephone sessions	UC	E, CB, R, C	Therapist	~6 weeks post‐baseline	Sexual bother •Patient Depression • Partner	Cohen's d 0.5 0.5
Chambers 2015	189	1. Peer‐delivered telephone support 2. Nurse‐delivered telephone counselling 8 (recruited pre‐surgery) or 6 (recruited post‐surgery) dyadic telephone sessions: 2 pre‐surgery and/or 6 post‐surgery over 22 weeks	UC	1. E, CB, PS, C 2. E, CB, SC, C, DS	PCa Nurse counsellor	12 months post‐recruitment	Use of ED tx Patient	*p* < 0.01
Couper 2015	62	Cognitive‐existential couple therapy 6 weekly dyadic face‐to‐face sessions	UC	CB, SC	Mental health professional	9 months post‐baseline	Relationship function Partner	*P* = 0.009
Giesler 2005 *Patient data only*	99	Post‐tx nursing support 6 monthly dyadic sessions; 2 face‐to‐face and 4 telephone sessions	UC	E, C	Oncology nurse	12 months post‐tx	Sexual limitation Cancer worry	*P* = 0.02 *P* = 0.03
Manne 2011	71	Intimacy‐Enhancing Therapy 5 dyadic face‐to‐face sessions over 8 weeks	UC	E, CB, SC, C	Therapist	8 weeks post‐ baseline	Cancer concern • Patient Cancer‐related distress • Partner Relationship satisfaction • Partner Intimacy • Partner	Sub‐group *P* = 0.02 Sub‐group *P* = 0.02 Sub‐group *P* = 0.0002 Sub‐group *P* = 0.001
Thornton 2004	80 patients, 65 partners	Pre‐surgical communication enhancement 1 dyadic face‐to‐face session	UC delivered by a nurse	SC, C	Trained counsellor	1 year post‐surgery	Stress Partner	partial η^2^ = 0.12
Titta 2006 *Patient data only*	57	Intracavernous injection‐focused sexual counselling for couples following patient training in PGE1‐intracavernous injections Six 3‐monthly dyadic face‐to‐face sessions	Control (partner invited to follow‐up visits every 3 months)	E, SC, C	NR	18 months post‐surgery	Erectile function Sexual satisfaction Sexual desire	*P* < 0.05 *P* < 0.05 *P* < 0.05
Walker 2013	27	Educational intervention for couples to maintain intimacy 1 dyadic face‐to‐face session + booklet	UC	E	Researcher familiar with ADT	6 months post‐enrolment	Intimacy •Patient Dyadic adjustment • Patient • Partner	Cohen's d 0.6 1.0 0.5

* Precision of effect and size of effect correspond to longest reported follow‐up; size of effect only reported if not significant. C, Communication; CB, Cognitive‐behavioural; DS, Decision Support; E, Education; ED, Erectile dysfunction; NS, Not significant; PCa, Prostate cancer; PS, Peer Support; QoL, Quality of Life; R, Relaxation; SC, Supportive Care; Tx, treatment; UC, Usual or standard care

**Table 3:** Inclusion of specific components in effective in *N* = 34 person‐focused interventions and *N* = 9 couple‐focused interventions

Components	Person‐focused interventions*	Couple‐focused interventions*
	% (n)	% (n)
Education	85% (29)	78% (7)
(psycho‐education, psycho‐sexual education, PCa education)		
Communication	44% (15)	78% (7)
(partner, sexual, health professional, general or type not specified)		
Peer support	41% (14)	11% (1)
(peer discussion, social support^)		
Cognitive‐behavioural	29% (10)	56% (5)
(cognitive restructuring, behaviour change, cognitive‐behavioural stress management)		
Decision support	24% (8)	11% (1)
(PCa treatment, sexual aids)		
Relaxation	24% (8)	11% (1)
(meditation, relaxation techniques)		
Supportive counselling	12% (4)	56% (5)
(counselling/psychotherapy, health professional discussion)		

* Note that some trials had multiple arms and more than one effective intervention.

^ Social support may include general group discussion with peers.

NB. Total percentages may exceed 100% because of multiple intervention components.

PCa, prostate cancer.

#### 3.5.1 Person‐focused

##### Decision making

Six trials improved patient decision‐making mostly for men diagnosed with early stage disease and/or recruited prior to treatment. Decision support, aid, or navigation reduced patient uncertainty,^31^, ^32^ conflict,^33^ and regret^34^, ^35^ about their treatment decision, and a combined online psycho‐educational intervention and moderated peer forum also reduced regret.^36^, ^37^ Patient self‐efficacy or confidence in their decision‐making was increased by decision navigation^34^ and interactive education interventions.^38^

##### Quality of life

An uncertainty management intervention improved QoL for patients on watchful waiting.^39^ In 2 trials, a 10‐week cognitive‐behavioural stress management intervention improved cancer‐specific QoL for patients with early stage disease.^40^, ^41^, ^42^

##### Fatigue

Participants who received Qigong^43^ or a health education intervention^44^, ^45^ experienced reduced fatigue.

##### Sexuality

Five trials reported better sexuality outcomes (80% of trials included majority of men who had radical prostatectomy). Combined education and group discussion,^46^, ^47^ and peer support,^48^ decreased sexual bother. A 10‐week group cognitive‐behavioural stress management intervention improved sexual function for men treated with prostatectomy (88% erectile dysfunction (ED)) who had high interpersonal sensitivity.^40^, ^41^ Sexual satisfaction improved for patients in a combined online psycho‐educational intervention and moderated peer support forum.^7^, ^36^ Only one trial improved multiple sexual outcomes; in addition to increased sexual QoL and orgasm satisfaction, Siddons et al^49^ reported increased masculine self‐esteem and sexual confidence for men treated with radical prostatectomy (90% ED) and who received a cognitive‐behavioural group intervention. Overall, 60% of trials reported follow‐up immediately following or close to intervention delivery.

##### Mental health

Eleven trials improved patient mental health outcomes. Patients receiving a combined online psycho‐educational intervention and moderated peer forum had less distress.^36^, ^37^ Qigong also decreased distress^43^; and a nurse‐led psycho‐education intervention^50^ and peer support^51^, ^52^ reduced depression. In 2 trials, a 10‐week cognitive‐behavioural stress management intervention improved emotional well‐being^53^ and depression.^23^

Mental health and cancer‐specific distress improved in younger, more highly educated patients who received a tele‐based psycho‐educational intervention.^54^ A multi‐modal intervention including cognitive‐behavioural therapy also reduced cancer‐related distress (avoidance) in patients with a monitor (cognitive scanning) coping style.^55^ Patients with high‐baseline depression or anxiety showed improvement in these constructs if they were allocated to either a multi‐modal intervention including either 30 or 90 minutes of an expert speaker/facilitated discussion.^56^, ^57^ In another trial, patients with lower baseline depression were less depressed if they received a combined education and group discussion intervention.^46^, ^47^ In this same study, patients with lower self‐esteem at baseline had less depression and better mental health if they participated in either a combined education and group discussion or education only intervention.

One trial improved patient and partner mental health outcomes.^44^, ^45^ Patients in the health education attention intervention had less depression, negative affect, stress, and greater spiritual well‐being. Effects on stress were more pronounced for men who were less educated, and greater reductions in depression were experienced if men were older, had lower PCa‐specific QoL, active chemotherapy, less social support or cancer knowledge. Patients receiving combined psychotherapy and education had more positive affect if they were more highly educated, had higher PCa‐specific QoL, or more social support. Partners in the health education intervention had improved depression, social, and spiritual well‐being.^44^, ^45^

#### 3.5.2 Couple‐focused

##### Quality of life

Intimacy‐enhancing therapy increased cancer‐specific QoL for patients with early stage disease and higher symptom‐related concerns at baseline.^26^

##### Sexuality

Four trials improved sexuality outcomes for patients only. Coping skills training reduced sexual bother,^58^ and intracavernous injection‐focused sexual counselling increased patient sexual function, sexual satisfaction, and desire.^59^ Post‐treatment nursing support lessened the extent to which sexual dysfunction interfered with spousal role activities.^60^ Prostate cancer nurse‐delivered and peer‐delivered telephone counselling interventions uniquely reported increased use of ED treatment at 12‐month post‐recruitment follow‐up for men with localised disease who had prostatectomy.^61^

##### Mental health

Mental health was improved in 5 trials, predominantly for partners. Coping skills training reduced partner's depressed mood.^58^ Pre‐surgical communication enhancement intervention reduced partner stress.^62^ Cancer‐related distress lessened in younger women receiving cognitive‐existential couple therapy,^63^ and partners with high levels of baseline distress receiving intimacy enhancing therapy.^26^ Cancer‐related worry also reduced for patients receiving post‐treatment nursing support.^60^

##### Relationships

Three trials improved relationship outcomes, mostly for partners. Cognitive‐existential couple therapy enhanced relationship function for female spouses.^63^ Intimacy enhancing therapy was associated with improved partner relationship satisfaction and intimacy for partners with lower baseline scores on these variables.^26^ Education to maintain intimacy also improved intimacy for patients starting ADT, and dyadic adjustment for patients and their female partners.^64^

### 3.6 Intervention delivery

Effective person‐focused interventions were most commonly delivered in an individual (53%) or group (47%) setting; face‐to‐face (50%), via telephone (26%) or online (26%); by a psychologist/counsellor (41%), nurse (29%) or self‐administered (26%). Couple‐focused interventions were delivered to dyads most commonly face‐to‐face (67%) or by telephone (44%); by a psychologist/counsellor (44%) or nurse (22%).

## 4 DISCUSSION

Psychosocial and psychosexual intervention can improve decision‐related distress, mental health, domain‐specific, and health‐related QOL in men with PCa. Combinations of educational, cognitive behavioural, communication, and peer support have been most commonly applied and found effective; followed by decision support and relaxation; and to a much lesser extent supportive counselling. These components were often used in a multi‐modal approach, and delivered through both face‐to‐face and remote technologies, with therapist, nurse or peer support. In sum, multi‐modal psychosocial and psychosexual care for men with PCa, particularly localised disease, is both acceptable and effective.

The evidence is less clear for the female partners of these men and couples as a dyadic unit. Couple‐focused interventions were the least acceptable approach and almost half of the couple interventions produced poorer outcomes for partners. When couple interventions were effective, they improved relationship outcomes for the partner but not the man; had a positive effect on the partner's mental health but conversely; improved sexuality outcomes for the man but not the partner. No interventions improved sexuality outcomes for female partners. Based on these results, effective and acceptable interventions for female partners and couples remain an area of uncertainty. It may be that couples interventions have been primarily focused on the PCa survivor's needs, leaving the partner's concerns poorly managed. This is an area where significant further work is required to understand the needs and preferences of couples, and to determine approaches to improve sexual and relationship satisfaction for both partners.

Limitations of the research to date include small sample sizes; low statistical power; suboptimal statistical methods in some studies; inconsistency in measurement approaches; a lack of diversity in participants—particularly with regards to gay and bisexual men; men with advanced PCa; and men from socio‐economically deprived; and non‐Anglo‐Saxon backgrounds. Long‐term survivorship outcomes (>2 years) are yet to be addressed. In addition, intervention components were often described in a vague way such that it was not always clear what was actually delivered; and treatment fidelity and therapist adherence was in most studies not well described. Strengths of the current review by comparison with previous reviews include a departure from a narrow focus on specific intervention type(s), single outcomes, or sub‐groups; a consideration of acceptability as well as statistical significance; and examination of not only intervention effectiveness but also who benefits by considering the influences of socio‐demographic and medical characteristics of men and their partners; intervention format and delivery; and acceptability.

### 4.1 Clinical implications

Standards for psychosocial care with regards to screening for distress are now widely accepted,^65^ and the validity of the distress thermometer for men with PCa is well established with clear cut‐offs for caseness.^11^ In this review, approximately one‐quarter of interventions reported effects moderated by socio‐demographic or psychosocial variables; with age, educational level, domain‐specific QOL, baseline mental health, and social support important considerations in designing care. Hence, as well as taking into account levels of distress, it is also important to consider factors that both moderate intervention effectiveness and place men at risk of greater psychosocial distress and poorer QOL (such as age, domain‐specific QOL, socio‐economic deprivation) over the longer term.^16^ Survivorship care plans for PCa will need to be stepped according to the type and depth of need.^66^, ^67^ In conclusion, there is sufficient evidence to recommend multi‐modal psychosocial and psychosexual interventions for men with PCa; with distress screening and risk and need assessment built in to tailor support to the individual. As yet, there is insufficient evidence to confirm the optimal approach for female partners and couples.

We note that in this review education and communication support was commonly applied effectively across both person and couples‐focused interventions. By contrast, supportive counselling was often used for couples, whereas for patients peer support was more common. This may reflect in part what support methods are acceptable to men. Care approaches also need to consider the impact of PCa on men's masculine identities and embed sensitivity to these masculinities in psychosocial and psychosexual interventions in a way that extends beyond a reductionist focus on erectile dysfunction.^65^

### 4.2 Future research

There is a need for improvement in the field in study quality, especially with regard to treatment fidelity. Where interventions are multimodal better clarity about therapy components would assist application by clinicians. There remain gaps in knowledge about effective interventions for men with advanced cancer and how to best help couples and partners warrants further investigation. Finally, expanded research is needed targeting the needs of gay and bisexual men and those from non‐Anglo‐Saxon and socio‐economically deprived backgrounds.

## CONFLICT OF INTEREST

The authors have no conflicts of interest to declare.

## APPENDIX A

### SEARCH STRATEGIES USED

**Table:** For Cochrane Central Register of Controlled Trials, Embase, MEDLINE, PREMEDLINE and PsycINFO, and MEDLINE Epub Ahead of Print databases (OVID):

#	Searches
1	exp Prostatic Neoplasms/
2	(prostat* adj3 (cancer* or carcinoma* or malig* or tumo?r* or neoplas* or metastas* or adeno*)).mp.
3	exp Neoplasms/
4	exp Prostate/
5	3 and 4
6	1 or 2 or 5
7	exp Affective Symptoms/
8	exp affective disorders/
9	affective disorders.mp.
10	exp Mood Disorders/
11	mood*.mp.
12	exp Depression/
13	depress*.mp.
14	exp Anxiety Disorders/
15	exp Anxiety/
16	anxiet*.mp.
17	anxious.mp.
18	exp Psychosomatic Medicine/
19	exp Stress, Psychological/
20	psycholog*.mp.
21	psychosoci*.mp.
22	(psycho adj soci*).mp.
23	(intrusive adj (thinking or thoughts)).mp.
24	intrusiveness.mp.
25	exp Mental Fatigue/
26	exp “Conflict (Psychology)”/
27	exp Emotions/
28	emotion*.mp.
29	unhapp*.mp.
30	happiness*.mp.
31	sad.mp.
32	sadness.mp.
33	(anhedon* or melanchol* or fear* or worr*).mp.
34	(stress* or distress* or nervous* or nervos*).mp.
35	(uncertainty or hope or wellbeing).mp.
36	well being*.mp.
37	exp Adaptation, Psychological/
38	exp Adjustment/
39	(cognitive adj3 adjustment).mp.
40	exp Decision Making/
41	decision making.mp.
42	decisional uncertainty.mp.
43	decisional regret.mp.
44	(decision* adj3 satisf*).mp.
45	exp Mental Health/
46	Behavioral Symptoms/
47	exp Attitude to Health/
48	exp Patient Satisfaction/
49	exp Personal Satisfaction/
50	((relationship or sexual) adj3 satisfaction).mp.
51	self efficacy.mp.
52	conflict*.mp.
53	(quality adj4 (life or living)).mp.
54	exp “Quality of Life”/
55	quality of life.mp.
56	(QOL or HRQOL).mp.
57	exp Social Support/
58	social support.mp.
59	Interpersonal Relations/
60	exp interpersonal relationships/
61	exp interpersonal interaction/
62	social interaction.mp.
63	exp Personal Autonomy/
64	autonomy.mp.
65	exp “independence (personality)”/
66	exp Fatigue/
67	(fatigue* or tiredness or libido* or impot*).mp.
68	exp Libido/
69	sex drive.mp.
70	erectile dysfunction.mp.
71	exp Sexual Dysfunction, Physiological/
72	exp Sexual Dysfunctions, Psychological/
73	exp Sexual Function Disturbances/
74	sexual dysfunction.mp.
75	exp Sexuality/
76	sexuality.mp.
77	exp Self Concept/
78	self image.mp.
79	(intimacy or wife or wives or dyad* or spous* or partner* or carer* or caregiv* or relational).mp.
80	exp marital relations/
81	or/7‐80
82	6 and 81
83	Randomized Controlled Trial.pt.
84	Pragmatic Clinical Trial.pt.
85	exp Randomized Controlled Trials as Topic/
86	“Randomized Controlled Trial (topic)”/
87	Randomized Controlled Trial/
88	Randomization/
89	Random Allocation/
90	Double‐Blind Method/
91	Double Blind Procedure/
92	Double‐Blind Studies/
93	Single‐Blind Method/
94	Single Blind Procedure/
95	Single‐Blind Studies/
96	Placebos/
97	Placebo/
98	(random* or sham or placebo*).ti,ab,hw.
99	((singl* or doubl*) adj (blind* or dumm* or mask*)).ti,ab,hw.
100	((tripl* or trebl*) adj (blind* or dumm* or mask*)).ti,ab,hw.
101	83 or 84 or 85 or 86 or 87 or 88 or 89 or 90 or 91 or 92 or 93 or 94 or 95 or 96 or 97 or 98 or 99 or 100
102	82 and 101
103	limit 102 to English language
104	limit 103 to yr = “2000‐current”

*Used Canadian Agency for Drugs and Technologies in Health filter for identifying randomised controlled trials (https://www.cadth.ca/resources/finding‐evidence accessed 17/02/2016)*

**Table:** For Health Technology Assessments (HTA) and Database of Abstracts of Reviews of Effects (DARE) databases (Ovid):

#	Searches
1	exp Prostatic Neoplasms/
2	(prostat* adj3 (cancer* or carcinoma* or malig* or tumo?r* or neoplas* or metastas* or adeno*)).mp.
3	exp Neoplasms/
4	exp Prostate/
5	3 and 4
6	1 or 2 or 5
7	exp Affective Symptoms/
8	exp affective disorders/
9	affective disorders.mp.
10	exp Mood Disorders/
11	mood*.mp.
12	exp Depression/
13	depress*.mp.
14	exp Anxiety Disorders/
15	exp Anxiety/
16	anxiet*.mp.
17	anxious.mp.
18	exp Psychosomatic Medicine/
19	exp Stress, Psychological/
20	psycholog*.mp.
21	psychosoci*.mp.
22	(psycho adj soci*).mp.
23	(intrusive adj (thinking or thoughts)).mp.
24	intrusiveness.mp.
25	exp Mental Fatigue/
26	exp “Conflict (Psychology)”/
27	exp Emotions/
28	emotion*.mp.
29	unhapp*.mp.
30	happiness*.mp.
31	sad.mp.
32	sadness.mp.
33	anhedon*.mp.
34	melanchol*.mp.
35	fear*.mp.
36	worry*.mp.
37	stress*.mp.
38	distress*.mp.
39	nervous*.mp.
40	nervos*.mp.
41	uncertainty.mp.
42	hope.mp.
43	wellbeing*.mp.
44	well being*.mp.
45	cope.mp.
46	coping.mp.
47	conflict.mp.
48	conflicts.mp.
49	exp Adaptation, Psychological/
50	exp Adjustment/
51	(cognitive adj3 adjustment).mp.
52	exp Decision Making/
53	decision making.mp.
54	decisional uncertainty.mp.
55	decisional regret.mp.
56	(decision* adj3 satisf*).mp.
57	exp Mental Health/
58	Behavioral Symptoms/
59	exp Attitude to Health/
60	exp Patient Satisfaction/
61	exp Personal Satisfaction/
62	((relationship or sexual) adj3 satisfaction).mp.
63	self efficacy.mp.
64	(quality adj4 (life or living)).mp.
65	exp “Quality of Life”/
66	quality of life.mp.
67	QOL.mp.
68	HRQOL.mp.
69	exp Social Support/
70	social support.mp.
71	Interpersonal Relations/
72	exp interpersonal relationships/
73	exp interpersonal interaction/
74	social interaction.mp.
75	exp Personal Autonomy/
76	autonomy.mp.
77	exp “independence (personality)”/
78	exp Fatigue/
79	fatigue.mp.
80	tiredness.mp.
81	exp Libido/
82	libido.mp.
83	sex drive.mp.
84	erectile dysfunction.mp.
85	impotence.mp.
86	exp Sexual Dysfunction, Physiological/
87	exp Sexual Dysfunctions, Psychological/
88	exp Sexual Function Disturbances/
89	sexual dysfunction.mp.
90	exp Sexuality/
91	sexuality.mp.
92	exp Self Concept/
93	self image.mp.
94	relational*.mp.
95	intimacy*.mp.
96	wife.mp.
97	wives.mp.
98	dyad*.mp.
99	spous*.mp.
100	partner*.mp.
101	exp marital relations/
102	carer*.mp.
103	caregiv*.mp.
104	or/7‐103
105	6 and 104

**Table:** For Allied and Complementary Medicine (AMED) database (OVID):

#	Searches
1	prostatic neoplasms/
2	(prostat$ adj5 (cancer$ or Neoplas$ or malignan$)).mp.
3	1 or 2
4	clinical trials/ or random allocation/
5	random$.mp.
6	trial.mp.
7	4 or 5 or 6
8	3 and 7
9	limit 8 to (English and yr = “2000‐Current”)

**Table:** For CINAHL database (EBSCO):

#	Searches
S17	S3 AND S15 Published date: 2009‐2016; English language; Exclude MEDLINE records
S16	S3 AND S15
S15	S4 OR S5 OR S6 OR S7 OR S8 OR S9 OR S10 OR S11 OR S12 OR S13 OR S14
S14	TX allocat* random*
S13	(MH “Quantitative Studies”)
S12	(MH “Placebos”)
S11	TX placebo*
S10	TX random* allocat*
S9	(MH “Random Assignment”)
S8	TX randomi* control* trial*
S7	TX ((singl* n1 blind*) or (singl* n1 mask*)) or TX ((doubl* n1 blind*) or (doubl* n1 mask*)) or TX ((tripl* n1 blind*) or (tripl* n1 mask*)) or TX ((trebl* n1 blind*) or (trebl* n1 mask*))
S6	TX clinic* n1 trial*
S5	PT Clinical trial
S4	(MH “Clinical Trials+”)
S3	S1 OR S2
S2	TX (prostat* N3 (cancer* OR carcinoma* OR malignan* or tumo#r* OR neoplas* OR metast* OR adeno*))
S1	(MM “Prostatic Neoplasms”)

*Used SIGN filter for identifying randomised controlled trials* (http://www.sign.ac.uk/methodology/filters.html#top accessed 17/02/2016)

## APPENDIX B

### FRAMEWORK FOR CATEGORISING PSYCHOSOCIAL INTERVENTION COMPONENTS

**Education**

- Psycho‐education: information or education about emotional impact of PCa and stress management; excludes cognitive‐behavioural approaches.
- Psycho‐sexual education: information or education about sexuality or psycho‐sexual impact of PCa or treatment.
- PCa education: information or education about PCa, treatment, and/or physical side effects.

**Cognitive‐behavioural**

- Cognitive restructuring: working with cognitions, challenging negative thoughts, refocusing thoughts onto positives.
- Behaviour change: Goal setting and problem solving or behavioural maintenance.
- Cognitive behavioural stress management: intervention identified as CBSM.

**Relaxation**

- Relaxation: meditation or relaxation techniques (eg, progressive muscle relaxation, Qigong, breathing exercises).

**Supportive counselling**

- Counselling/psychotherapy (as identified by the study author): counselling or therapy offered as part of the intervention including sexual therapy, excludes cognitive‐behavioural approaches.
- Health professional discussion: discussion with a health professional (excludes counselling/psychotherapy, routine/standard care).

**Peer support**

- Peer support: shared experience with a peer who also has PCa (includes support groups, social support).
- Social support: mentions social support generally and may also include informal peer support in a group setting, or does not specify type.

**Communication**

- Partner: information or skill development to promote partners/couples communication (eg, treatment side‐effects, intimacy), excludes communication about sex.
- Sexual: information or skill development to enable communication with partner about sex.
- Health professional: information or skill development to encourage communication with health professional regarding treatment or post‐treatment concerns (eg, side‐effects).
- Communication: general interpersonal communication or communication unspecified.

**Decision support**

- PCa treatment: decision aid, tool or navigator to support PCa treatment decision.
- Sexual aids: decision aid, tool or navigator to support decision to use erectile or other sexual aid or treatment.

## APPENDIX C

### RISK OF BIAS ASSESSMENT OF TRIALS ADDRESSING QUESTION 1 (PATIENTS *N* = 56 TRIALS) AND QUESTION 2 (PARTNERS *N* = 14 TRIALS)

**Table:**

Risk of bias category	Q1 *N* (%)	Q2 *N* (%)
1. What was the risk of bias from the random sequence generation?		
Low: Adequate (eg, computer random number generator)	20 (36)	5 (36)
High: Inadequate	2 (4)	0 (0)
Unclear: Not reported	34 (61)	9 (64)
2. What was the risk of bias from the allocation concealment?		
Low: Adequately concealed (eg, central randomisation)	16 (29)	3 (21)
High: Inadequately concealed (eg, sealed envelopes)	0 (0)	0 (0)
Unclear: Concealment not reported or insufficient information to permit judgement	40 (71)	11 (79)
3. What was the risk of bias from incomplete outcome data^a^?		
Low: Loss to follow‐up less than 50% and balanced across arms (<5% difference)	19 (34)	4 (29)
High: Loss to follow‐up greater than 50% or not balanced between arms or non ITT analyses	24 (43)	6 (43)
Unclear: Insufficient information to permit judgement	13 (23)	4 (29)
4. What was the risk of bias from selective outcome reporting?		
Low: Study protocol available and all pre‐specified outcomes reported	7 (13)	3 (21)
High: Study protocol available and not all pre‐specified outcomes reported	14 (25)	6 (43)
Unclear: Insufficient information to permit judgement (eg, study protocol not found)	35 (63)	5 (36)
5. What was the risk of bias from other sources**, a?		
Low: Study appears free of other sources of bias	39 (70)	12 (86)
High: There is at least one important risk of bias from other sources	14 (25)	2 (14)
Unclear: Insufficient information to assess whether there is a risk of bias from other sources	3 (5)	0 (0)

a For primary outcome

** Including differences in disease stage or follow‐up between arms, and analyses that did not consider baseline measures

ITT, intention‐to‐treat

## APPENDIX D

### ELIGIBLE TRIALS INCLUDED IN THE REVIEW ADDRESSING QUESTION 1 (PATIENTS) AND QUESTION 2 (PARTNERS)

**Table A1:** Trials comprising person‐focused interventions (*N* = 43: 41 patient only, 1 partner only, 1 patient and partner)

Study	Participants #	Intervention	Intervention components	Comparator	Relevant outcomes	Precision of effect *	Size of effect *	Key findings	Acceptability
Ames 2011 USA	57 men with biochemical recurrence Median age 76 years	Multi‐modal intervention which included psychosocial components Delivered by clinical psychologist, medical oncologist, dietician and physiatrist 8 group face‐to‐face sessions over 8 weeks Follow‐up 6 months post‐intervention	E, CB, R, PS	Wait‐list control	Mental health PCa‐related anxiety Stress Mood PCa‐related QoL	NR NR NR NR NR	−0.0 0.2 0.0 −0.1 0.1	The multi‐modal intervention did not significantly (or with a moderate or large effect size) improve outcomes	100% retention at end of intervention 97% participants attended ≥6 of 8 intervention sessions 80% rated on a 5‐point scale helpfulness of intervention as 4 (very much) or 5 (extremely)
Badger 2011, 2013 USA *Patients + partners*	71 men and social network members (93% female; 83% partner, 13% family member, 4% friend) Men ≤6 months since tx Minimum 11% stage IV Patient M age 67 years; Partner M age 61 years	1. Interpersonal psychotherapy + cancer education for patient and partner Delivered by nurse or social worker Patients: 8 individual telephone sessions over 8 weeks Partners: 4 individual telephone sessions over 8 weeks Follow‐up 8 weeks post‐intervention	1. E, SC, PS, C 2. E	2. Health education attention condition for patient and partner Delivered by research assistants Patients: 8 individual telephone sessions over 8 weeks Partners: 4 individual telephone sessions over 8 weeks	*Patients* Depression Positive affect Negative affect Stress Fatigue PCa‐related QoL Social well‐being Spiritual well‐being	*P* < 0.001 NS *P* < 0.001 *P* < 0.001 *P* < 0.01 NS NS *P* < 0.01	NR NR NR NR NR NR NR NR	The health education attention intervention significantly improved depression, negative affect, stress, fatigue, and spiritual well‐being when compared with psychotherapy + education intervention Men in the psychotherapy + education intervention had significantly greater improvement in positive affect if they were more highly educated, had higher PCa‐specific QoL or had more social support from friends Men in the health education intervention had significantly greater reduction in depression if they were older, had lower PCa‐specific QoL, were on active chemotherapy, had less social support or less cancer knowledge Men in the health education intervention had significantly greater reduction in stress if they were less educated	40% recruitment rate 6% withdrew from psychotherapy + education intervention and 9% withdrew from education attention intervention 86% attendance in psychotherapy + education arm; 89% attendance in education attention intervention
					*Partners* Depression Positive affect Negative affect Stress Fatigue Social well‐being Spiritual well‐being	*P* < 0.05 NS NS NS *P* < 0.01 *P* < 0.01 *P* < 0.01	NR NR NR NR NR NR NR	The health education attention intervention significantly improved depression, fatigue, social, and spiritual well‐being when compared with psychotherapy + education intervention	
Bailey 2004 USA	39 men ≤10.3 years post‐tx decision on watchful waiting Stage T1‐3 (2% T3) M age 75 years	Uncertainty management: cognitive reframing tailored to patient needs Delivered by a nurse 5 weekly individual telephone sessions Follow‐up ~5 weeks post‐intervention	E, CB, C, DS	Usual care	QoL (Cantrill's ladder) Mood	*P* = 0.01 NS	NR NR	Uncertainty management intervention significantly improved QoL when compared with usual care	76% recruitment rate 5% withdrew from intervention 95% follow‐up in both arms
Beard 2011 USA	54 men undergoing radiotherapy 91% ADT Stage M0 Median age 64 years	Relaxation response therapy with cognitive restructuring (RRT) Delivered by psychologist 8 weekly individual face‐to‐face sessions during radiotherapy period Follow‐up 8‐12 weeks post‐intervention	CB, R	1. Wait‐list control 2. Reiki therapy	Anxiety Depression Cancer‐related QoL Emotional well‐being subscale	NS NS NS NS	NR NR NR NR	No significant improvements in outcomes were found when all 3 arms were compared	73% recruitment rate 100% in Reiki and RRT arms completed study 89% in RRT arm attended all 8 sessions
Berglund 2007 Sweden	211 men ≤6 months since dx Stage 20% M1 M age 69 years	1. Physical training + relaxation 2. Information sessions 3. Physical training + information sessions + relaxation Psychosocial components for all interventions delivered by physiotherapist (1, 3), nurse and urologist/oncologist (2, 3) All interventions comprised 7 group face‐to‐face sessions over 7 weeks Follow‐up 12 months	1. R 2. E, PS 3. E, R, PS	Standard care	Anxiety Depression	NS NS	NR NR	The multi‐modal interventions did not significantly improve outcomes	50% recruitment rate 8% withdrew from physical training and physical training + information arms; 7% withdrew from information only arm—primarily because of transport issues
Berry 2012, 2013 USA	494 men recently dx and pre‐tx (50% had tx preference at baseline) Stage T1‐2 Median age 62‐63 years	Decision support system Self‐administered 1 individual internet session Follow‐up 6 months post‐intervention	E, C, DS + Clinic's usual educational resources (eg, pamphlets and links to reputable websites)	Usual care	Decisional uncertainty (100 unit scale) Decisional satisfaction Decisional regret *Subgroup of men who made decision by 6 months* Total decisional conflict (100 unit scale)	*P* = 0.04 NS NS NS	Coefficient −3.61 units NR NR −1.75 units	Internet decision support significantly reduced decisional uncertainty when compared with usual care	68% recruitment rate 100% compliance Authors identified good acceptability (M 25.1 on scale of 6‐30)
Campo 2014 USA	40 men <26 years since dx with significant fatigue and sedentary 48% ADT 61% Stage III‐IV Median age 72 years	Qigong Delivered by qigong Master and his certified instructors 24 twice weekly group face‐to‐face sessions Follow‐up 1 week post‐intervention	R	Stretch control (24 twice weekly group face‐to‐face sessions)	Fatigue (scale 0‐52) Distress	*P* = 0.02 *P* = 0.002	Cohen's d NR ≥ 3‐point improvement in fatigue score for 69% qigong vs 38% controls −1.2	Qigong significantly improved fatigue and reduced distress when compared with stretch control however 47% had advanced disease in qigong arm compared with 82% in stretch control arm	18% consented to eligibility assessment 20% withdrew from qigong arm; 35% withdrew from stretch control arm 85% median rate of attendance for qigong arm; 43% for stretch control
Carmack‐Taylor 2006, 2007 USA	134 men on ADT for next 12 months M age 69 years 12% depressed requiring clinical evaluation	1. CB training to increase physical activity +30 minutes of expert speaker or facilitated discussion 2. 90 minutes of expert speaker or facilitated discussion All interventions delivered by a group facilitator who was supervised by a licenced clinical psychologist All interventions comprised 21 group face‐to‐face sessions over 6 months Follow‐up 6 months post‐intervention	1. E, PS 2. E, PS	Standard care	Mental health Anxiety Depression Self‐esteem	NS NS NS NS	NR NR NR NR	For the outcomes of depression and anxiety, there were significant group x baseline level interactions indicating that men with high rather than low baseline levels of depression (*P* = 0.02) or anxiety (*P* = 0.002) were more likely to benefit from either of the 2 interventions	64% recruitment rate 4% *90 minutes E + PS* and 3% controls withdrew 70% mean attendance rate for *90 minutes E + PS;* ~82% attended at least 50% of sessions
Chabrera 2015 Spain	142 men with localised disease pre‐tx M age 69 years	Decision aid Self‐administered Individual printed Follow‐up 3 months post‐baseline	E, C, DS	Usual care	Decisional conflict	*P* < 0.001	Difference in change from baseline score −24.4 (100‐point scale)	Decision aid significantly reduced decisional conflict when compared with usual care	100% recruitment of eligible men 84% intervention and 82% control had follow‐up
Chambers 2013 Australia	740 men with localised disease pre‐tx M age 63 years	Telephone psycho‐educational intervention Delivered by nurse counsellors 5 individual telephone sessions: 2 pre‐tx, and at 3 weeks, 7 weeks and 5 months post‐tx Follow‐up 24 months post‐tx	E, CB, R, DS	Usual care	Cancer‐specific distress Decisional uncertainty PSA anxiety Mental health Well‐being Sexual bother	NS NS NS NS NS NS	NR NR NR NR NR NR	For a subgroup of participants who were younger with higher education levels, the psycho‐educational intervention significantly improved mental health (*P* = 0.04) and cancer‐specific distress (*P* < 0.008)	82% recruitment rate At 6 months post‐tx, 7% withdrawn in intervention arm; 5% withdrawn in control arm 100% median rate of intervention attendance
Chambers 2017 Australia	189 men with metastatic disease and/or castration‐resistant biochemical progression 99% had received ADT M age 71 years 40% significant baseline distress	1. Mindfulness‐based cognitive therapy (MBCT) Delivered by health professionals with oncology experience and professional training in MBCT 8 weekly group teleconference sessions Follow‐up 9 months post‐baseline	1. E, CB, R, PS 2. E	2. Minimally enhanced usual care Self‐administered Individual CD and information	Psychological distress Cancer‐specific distress PSA anxiety PCa‐specific QoL	NS NS NS NS	NR NR NR NR	MBCT did not significantly improve outcomes compared with minimally enhanced usual care	46% recruitment rate 14% withdrew from MBCT arm and 6% withdrew from minimally enhanced usual care arm 30% attended all 8 MBCT sessions 72% of 61 men who completed a satisfaction survey rated intervention as very to extremely helpful
Daubenmier 2006; Frattaroli 2008 USA	93 men on active surveillance Stage T1‐T2 M age 66 years	Multi‐modal lifestyle intervention including 1 hour/day stress management practice Deliverer of intervention NR Introduced at 1‐week residential retreat Weekly group face‐to‐face sessions ongoing Follow‐up 24 months post‐baseline	R, PS	Usual care	Mental health Stress Sexual function	NS NS NS	NR NR NR	The multi‐modal intervention did not significantly improve outcomes	51% recruitment rate Mean self‐reported programme adherence 95% at 24 months 91% intervention and 86% control completed 12‐month post‐baseline assessments
Davison 2007 Canada	324 men recently dx and considering tx Stage T1‐T2 M age 62 years	1. Individualised decision support Self‐administered 1 individual interactive computer session Follow‐up 4‐6 weeks post‐baseline (after decision made)	1. E, DS 2. E	2. Generic decision support Self‐administered 1 individual video session	Decisional conflict	NS	NR	Individualised decision support intervention did not significantly improve decisional conflict when compared with generic decision support	86% recruitment rate 100% compliance 91% individualised intervention and 90% generic intervention post‐intervention follow‐up Mean total rating of satisfaction with preparation in decision making was 2.80 for individualised arm and 2.67 for generic arm. The individualised intervention was rated higher in helping considering pros and cons and communicating opinions
Diefenbach 2012 USA	91 men 4‐6 weeks since dx who had not made a tx decision Stage T1‐T2 M age 62 years	1. Prostate Interactive Educational System (PIES) with or without tailoring to patient's information seeking style *(combined results from both PIES arms)* Self‐administered 1 individual internet/CD‐ROM session Follow‐up immediately post‐intervention	1. E, DS 2. E	2. Control Asked to read Standard National Cancer Institute booklets on PCa for 45 minutes Self‐administered 1 individual booklet	Confident about tx choice Prefer more time to decide Prefer more information Feel informed	*P* = 0.02 NS *P* = 0.02 NS	NR NR NR NR	The interactive education intervention improved confidence about tx choice and reduced preference for more information when compared with printed information (however, baseline levels of confidence about tx choice were not measured)	75% recruitment rate 100% compliance 82% PIES with tailoring, 75% PIES without tailoring and 79% controls had post‐intervention follow‐up Mean rating of helpfulness in decision making was 4.29 for tailored PIES, 4.10 for non‐tailored arm and 1.79 for control, scored 1 (not at all) to 5 (very much)
Dieperink 2013 Denmark	161 men 4 weeks since radiotherapy Stage T1‐T3 (46% T3) M age 68‐69 years	Individualised psychosocial (2 sessions) and physical therapy (2 sessions) counselling Psychosocial components delivered by radiation therapists 2 individual psychosocial face‐to‐face sessions over 12‐14 weeks Follow‐up 22 weeks post‐baseline	SC	Usual care	Mental health Sexual QoL	NS NS	NR NR	The multi‐modal intervention did not significantly improve outcomes	77% recruitment rate 3% withdrew from intervention; 2% withdrew from control 90% had 100% attendance rate
Feldman‐Stewart 2012 Canada	156 men with a new dx and making a tx decision Stage T1‐T2 60% ≥ 60 years	1. Decision aid—Information + explicit values clarification exercises Self‐administered 1 individual computerised session Follow‐up 12‐18 months post‐decision	1. E, DS 2. E	2. Decision aid—Information only Self‐administered 1 individual computerised session	Decision regret	NS	NR	Including values clarification exercises in a decision aid did not significantly improve decision regret when compared with a decision aid providing information only	37% recruitment rate (refusal because of: knowing what tx preferred or not needing further resources/help) 100% intervention completion and immediate post‐intervention follow‐up
Hack 2007 Canada	425 men attending primary tx consultation with radiation oncologist Stage T1‐4 (15% T3‐4) M age 67 years	Audiotape of tx consultation with radiation oncologist Individual audiotape Follow‐up 12 weeks post‐consultation	E, DS	No audio‐tape of tx consultation	PCa‐related QoL Mood	NS NS	NR NR	An audiotape of radiotherapy tx consultation did not significantly improve outcomes	96% recruitment rate 35% of those who received tape did not listen to it M 83.0 for patients who listened to the tape (0 extreme dislike‐100 extreme like); 47% rated it ≥75
Hacking 2013 UK	123 men newly dx with localised or early stage disease considering tx options and referred to urologist M age 65‐67 years	Decision navigation Delivered by research assistants 1 individual face‐to–face or phone session, audiotape and written notes Follow‐up 6 months post‐consultation	DS	Usual care	Decisional self‐efficacy Decisional conflict Decisional regret Anxiety Depression Mental adjustment to cancer: Fighting spirit Anxiety Fatalism	*P* = 0.009 NS *P* = 0.04 NS NS NS NS NS	NR NR NR NR NR NR NR NR	Decision navigation significantly increased decisional self‐efficacy and reduced decision regret when compared with usual care	43% recruitment rate 2% withdrew from intervention prior to medical consultation At 6 months, men in the intervention arm used the consultation plan M 3.3 times, the consultation summary M 3.1 times and listened to the audiotape M 2.4 times 92% of respondents rated the intervention as very helpful before the urologist consultation
Huber 2013 Germany	203 men attending pre‐prostatectomy consultation M age 63 – 64 years	Multimedia‐supported pre‐operative education Delivered by physician 1 individual computer‐based session Follow‐up 6‐10 hours after pre‐operative education	E	Standard pre‐operative education Delivered by physician	Anxiety Decisional confidence	NS NS	Difference −0.5 −0.3	The addition of multimedia‐support to standard pre‐operative education did not significantly improve outcomes	96% recruitment rate 100% compliance Complete satisfaction with pre‐operative education reported by 69% intervention and 52% control (*P* = 0.016)
Kim 2002 USA	152 men undergoing radiotherapy Stage A‐C (21% stage C) M age 71 years	Specific information about radiotherapy procedures and side effects Self‐administered Individual audiotapes of 2 information sessions Follow‐up at end of radiotherapy tx	E	General information about radiotherapy Self‐administered Individual audiotapes of 2 information sessions	Negative affect Fatigue	NS NS	NR NR	Providing specific information did not significantly improve outcomes when compared with providing general information	Cannot assess
Lepore 2003; Helgeson 2006 USA	250 men ≤1 month since tx started Stage T1‐3 (12.8% T3) M age 65‐66 years	1. Education + group discussion (attended with a family member or friend) Education delivered by urologist, oncologist, dietician, oncology nurse and clinical psychologist Group discussion delivered by male clinical psychologist to patients and by female oncology nurse to female family members 6 weekly group face‐to‐face sessions 2. Education only Delivered by urologist, oncologist, dietician, oncology nurse and clinical psychologist 6 weekly face‐to‐face group sessions Follow‐up 12 months post‐ intervention	1. E, PS 2. E	Standard medical care	Mental health Depression Sexual function Sexual bother	NS NS NS *P* < 0.01	NR NR NR NR	Education and group discussion intervention significantly reduced sexual bother when compared with standard care For depression, there was a significant group x self‐esteem interaction indicating that men with lower self‐esteem were more likely to benefit from either intervention and a significant group x baseline depression interaction indicating that men with lower baseline depression levels were likely to benefit from education + group discussion intervention (*P* < 0.05) For mental health, there was a significant group x self‐esteem interaction indicating that men with lower self‐esteem were more likely to benefit from either intervention (*P* < 0.05)	85% consented to assessment for eligibility; 77% of those eligible agreed to participate 67% mean attendance rate in both intervention arms Helpfulness M 4.22 (scored 1 not at all to 5 very)
Manne 2004 USA *Partners only*	60 female partners of men dx with any stage of PCa (5% Stage IV) M age 60 years 18% clinically significant distress (MHI score > 1.5 SD > normative mean) 49% had IES score > 19, ie, high cancer‐related distress	Psychosocial educational groups for wives/partners Delivered by radiation oncologist, nutritionist, clinical psychologists and social worker 6 weekly group face‐to‐face sessions Follow‐up 1 month post‐ intervention	E, CB, R, C	Standard psycho‐social care Support from a social worker and referral to a community mental health professional	Distress Cancer related‐distress Relationship communication about cancer	NS NS NS	NR NR NR	Psychosocial education groups did not significantly improve outcomes when compared with standard psychosocial care	57% recruitment rate (refusal because of: distance from centre, time and health problems) 11% drop‐out from intervention and 9% from control 85% mean attendance rate for intervention
McQuade 2016 USA	66 men scheduled to undergo radiotherapy Stage I‐III (21% ≥ T3a) M age 65 years	Qigong/Tai chi Delivered by trained qigong master 3 individual or group face‐to‐face sessions per week during radiotherapy (6‐8 weeks) Follow‐up 3 months post‐radiotherapy	R	1. Light exercise Delivered by exercise physiologist 3 individual or group face‐to‐face sessions per week during radiotherapy (6‐8 weeks) 2. Wait‐list control	Fatigue	NS	NR	A qigong and tai chi programme during radiotherapy did not significantly improve fatigue when compared with a light exercise programme or usual care	38% recruitment rate 81% intervention, 73% light exercise control and 92% wait‐list control had follow‐up at end of intervention
Mishel 2002 USA *Reported patient data only*	252 couples (% female partner unclear) Men ≤2 weeks since catheter removal following surgery or ≤3 weeks since radiotherapy start Stage T1‐3 (27% T3) Patient M age 64 years	1. Uncertainty management—Patient only Delivered by nurse 8 weekly individual phone calls 2. Uncertainty management—Patient and support person Delivered by nurse 8 weekly individual (not dyad) phone calls Follow‐up 7 months post‐baseline	1. E, CB, C 2. E, CB, C	Usual care	Illness appraisal/ uncertainty Symptom intensity Symptom number Sexual function Sexual satisfaction	NS NS NS NS *P* = 0.02	NR NR NR NR NR	For patients, sexual satisfaction was significantly different between arms over time however actual effects of uncertainty management intervention were unclear	77% recruitment rate
Mishel 2009 USA *Reported patient data only*	252 couples (~80% married or partnered) Men 10‐14 days pre‐tx consultation Stage T1‐2b Patient M age 63 years	1. Decision navigation—Patient only Information + telephone calls to review content, identify/ formulate questions and practise skills Phone calls delivered by nurse Individual self‐administered booklet, DVD and 4 phone calls over 7‐ 10 days 2. Decision navigation—Patient and primary support person Intervention as for patient only intervention delivered to both patient and their support person individually (not dyad) Phone calls delivered by nurse Individual/couple self‐administered booklet, DVD and 4 phone calls over 7‐10 days Follow‐up 3 months post‐baseline	1. E, SC, C, DS 2. E, SC, C, DS	Control Handout on staying healthy during tx	Mood Well‐being Decisional regret	NS NS *P* = 0.01	NR NR NR	Patients in both decision navigation interventions had significantly lower decision regret scores than controls	75% recruitment rate Helpfulness of information resources rated significantly (*P* < 0.05) higher for men in either tx group vs controls
Osei 2013 USA	40 men ≤5 years since dx M age 67 years	1. Online support Us TOO International website Self‐administered 3 times per week individual internet sessions over 6 weeks Follow‐up 8 weeks post‐baseline	1. E, PS 2. E	2. Resource kit US TOO International pamphlets Self‐administered Individual booklet over 6 weeks	Mental health Sexual QoL Life satisfaction (Well‐being) Relationship satisfaction Positive Negative	NS NS NS NS NS	NR NR NR NR NR	Online support and information did not significantly improve outcomes when compared with printed information	5% of patients who received invitation were interested and eligible 58% said online support community met all or most of their needs M satisfaction 3.01 (scale 1‐4)
Parker 2009; Gilts 2013 USA	159 men scheduled for prostatectomy Stage I‐III (12.6% stage III) M age 60‐61 years	1. Pre‐surgical stress management sessions Delivered by clinical psychologist 4 individual face‐to‐face sessions (3 prior to surgery and 1 at 48 hours post‐surgery + printed materials + audiotape 2. Supportive attention Delivered by clinical psychologist 4 individual face‐to‐face sessions Follow‐up 12 months post‐surgery	1. E, CB, R, SC, PS 2. SC	No meetings with a clinical psychologist	Mood Cancer‐related distress Mental health Sexual function Sexual bother *Subgroup with all measures at baseline* and *12 months* Distress Marital relationship satisfaction	NS NS NS NS NS NS NS	NR NR NR NR NR NR NR	Stress‐management and supportive care interventions did not significantly improve outcomes when compared with controls	77% recruitment rate 58% stress management arm, 72% supportive attention arm and 69% controls had 6 weeks post‐surgery follow‐up
Penedo 2006; Molton 2008 USA	191 men <18 months since tx Stage T1‐T2 M age 65 years	1. 10‐week group CB stress management techniques + relaxation training Co‐delivered by licenced clinical psychologist and/or master's level clinical psychology students 10 weekly group face‐to‐face sessions Follow‐up 12‐13 weeks post‐baseline	1. E, CB, R, PS, C 2. E	2. Half‐day seminar on stress management techniques Same content as 10‐week intervention Co‐delivered by licenced clinical psychologist and/or master's level clinical psychology students 1 group face‐to‐face session	Cancer‐related QoL Follow‐up 12‐13 weeks post‐baseline *Subgroup + additional participants* *121 men who had undergone prostatectomy* *88% significant ED* *M age 60 years* Sexual function	*P* < 0.05 *P* < 0.05	NR NR	Stress management training delivered as 10 weekly sessions significantly improved cancer‐related QoL when compared with a single half‐day intervention For men who had undergone a prostatectomy, the 10‐week intervention significantly improved sexual function compared with the half‐day intervention particularly for men with high interpersonal sensitivity However, there was a difference in assessment for the 10‐week intervention (assessed 2‐3 weeks post‐intervention) and the half‐day seminar (assessed 7‐8 weeks post‐seminar)	56% recruitment rate for eligible men 8% withdrew from 10‐week intervention 6% withdrew from half‐day intervention 79% 10‐week arm and 84% half‐day arm completed post‐ intervention follow‐up
Penedo 2007 USA	93 monolingual Spanish speaking men <21 months since tx Stage T1‐T2 M age 66 years	1. 10‐week culturally sensitive group CB stress management techniques + relaxation training Co‐delivered in Spanish by licenced clinical psychologist and clinical health psychology graduate student 10 weekly group face‐to‐face sessions Follow‐up 12‐13 weeks post‐baseline	1. E, CB, R, PS, C 2. E	2. Half‐day culturally sensitive seminar on stress management techniques Same content as 10‐week intervention Co‐delivered In Spanish by licenced clinical psychologist and clinical health psychology graduate student 1 group face‐to‐face session	Cancer‐related QoL Sexual QoL	*P* = 0.006 NS	NR NR	Stress management training delivered as 10 weekly sessions significantly improved cancer‐related QoL when compared with the half‐day stress management training session	37% recruitment rate for eligible men 9% withdrew from 10‐week intervention 3% withdrew from half‐day intervention 77% 10‐week arm and 75% half‐day arm completed post‐intervention follow‐up
Petersson 2002 Sweden	118 men (~ 50% on watchful waiting) ≤ 3 months since dx M age 71 years	Randomly assigned to +/‐ individualised intervention including CB therapy Group rehabilitation programme (only or + individual support) which included psychosocial components + physical activity Psychosocial components delivered by oncologist, urologist/surgeon and dietician (education), psychologist and oncology nurse (CBT) and physiotherapist (relaxation) 8 group face‐to‐face sessions over 8 weeks + booster group session after 2 months + written information Follow‐up 3 months post‐intervention start	E, CB, R	No group intervention	Anxiety Depression Cancer‐related distress Intrusion Avoidance	NS NS NS NS	NR NR NR NR	For the outcome of avoidance there was a significant group x coping style interaction indicating that men with monitor (cognitive scanning) rather than blunter (cognitive avoidance) coping style were more likely to benefit from the multi‐modal intervention (*P* < 0.01)	61% in group arm and 68% in no group arm had post‐intervention follow‐up
Schofield 2016 Australia	331 men starting radical radiotherapy 47% high risk 31% pre‐baseline ADT 39% salvage EBRT M age 67‐68 years	Nurse‐led group psycho‐educational consultation intervention Delivered by uro‐oncology nurses 4 x group face‐to‐face sessions Sessions at beginning of radiotherapy, mid‐radiotherapy, radiotherapy completion, and 6 weeks post‐radiotherapy +1 individual session after week 1 group consultation Follow‐up 6 months post‐radiotherapy	E, PS, C	Usual care	Anxiety Depression Distress Sexual QoL Sexuality needs	NS *P* = 0.0009 NS NS NS	Effect size 0.0 0.1 0.1 0.1 0.0	Psycho‐educational intervention significantly reduced rise in depression when compared with control arm	71% recruitment rate 3% withdrew from intervention 68% attended all 4 intervention group sessions
Siddons 2013 Australia	60 men 6‐60 months since prostatectomy, 90% ED Stage M0 PSA < 0.1 ng/mL M age 62 years 13% moderate‐severe stress, 10% moderate‐severe anxiety, 10% moderate‐severe depression	CB group intervention Delivered by psychologist 8 group face‐to‐face sessions over 8 weeks Follow‐up 8 weeks (end of intervention)	E, CB, R, C	Wait‐list	Masculine self‐esteem Sexual confidence Marital satisfaction Sexual QoL Sexual function Sexual cognition Sexual arousal Sexual behaviour Orgasm satisfaction Drive/relationship	*P* = 0.037 *P* = 0.001 NS *P* = 0.046 NS NS NS *P* = 0.047 NS	NR NR NR NR NR NR NR NR NR	CB intervention significantly improved masculine self‐esteem, sexual confidence, sexual QoL and orgasm satisfaction when compared with wait‐list control	7% recruitment rate (did not participate because of not feeling in need of psychological support, work commitments, difficulties commuting) 100% intervention and control had follow‐up at end of intervention
Stefanopoulou 2015 UK	68 men receiving ADT with problematic hot flushes and night sweats (HFNS) Stage 31% M1 M age 69 years 25% > cut‐off for depression 21% > cut‐off for anxiety	Guided self‐help CB therapy Self‐administered 4‐week individual intervention (booklet and CD) with 1 telephone call at 2 weeks for support and guidance delivered by a clinical psychologist Follow‐up 32 weeks post‐randomisation	E, CB, R, SC	Usual care	Depression Anxiety Cancer‐related QoL	NS NS NS	Adjusted mean difference −0.52 −0.32 −0.97	CB therapy did not significantly improve outcomes when compared with usual care	75% recruitment rate Compliance: 88% read either all (69%) or more than half of booklet (19%) 79% used relaxation CD and 76% practised paced breathing at least once a week 97% of both intervention and controls had follow‐up at end of intervention
Taylor 2010 USA	120 men with a new dx prior to tx decision Stage T1‐T2 M age 65 years	1. Decision aid—Information +3 interactive decision tools Self‐administered Individual CD‐ROM Follow‐up 1 month post‐baseline	1. E, DS 2. E	2. Decision aid—Information only Self‐administered Individual CD‐ROM	Mental health Sexual function Sexual bother Decisional conflict	NS NS NS NS	NR NR NR NR	Including interactive decision tools in a decision aid did not significantly improve outcomes when compared with a decision aid providing information only	86% recruitment rate (refusal because of: 9% lack of interest, 3% no need for further information, 2% uncomfortable with computers) 69% information + decision tool intervention used CD – 42% accessed all 3 decision tools 90% information only intervention used CD 88% of information + decision tool and 89% of information only had follow‐up at end of intervention Mean rating of helpfulness of CD‐ROM for both arms combined was 60.4 on 0‐100 scale (No association found between helpfulness rating and group)
Templeton 2004 UK	58 men tx with ADT 42% aged 71‐80 years	Nurse delivered education booklet Participant read booklet with urology nurse Delivered by urology nurse Single individual face‐to‐face session Follow‐up 1 month post‐baseline	E	Usual care	Prostate cancer‐related QoL	NR	NR	NR (no comparative results reported)	89% recruitment rate 100% compliance 97% intervention and 93% controls had follow‐up
Traeger 2013 USA	257 Spanish speaking men <18 months since tx Stage T1‐T2 M age 65 years	1. 10‐week group CB stress management techniques + relaxation training with culturally sensitive seminars Co‐delivered by clinical psychologist and clinical psychology graduate 10 weekly group face‐to‐face sessions Follow‐up 12‐13 weeks post‐baseline	1. E, CB, R, PS, C 2. E	2. Half‐day seminar on stress management techniques with culturally sensitive seminars Same content as 10‐week intervention Co‐delivered by clinical psychologist and clinical psychology graduate 1 group face‐to‐face session	Emotional well‐being	*P* < 0.05	NR	Stress management training delivered as 10 weekly session significantly improved emotional well‐being when compared with a single half‐day stress management training session	52% recruitment rate 14% withdrew from 10‐week intervention 8% withdrew from half‐day intervention 82% 10‐week arm and 84% half‐day arm completed post‐ intervention follow‐up
Van Tol‐Geerdink 2013, 2016 Netherlands	240 men who had not made a tx decision Stage T1‐T3a (<12% T3) M age 64 years	Decision aid Delivered by a researcher 1 individual face‐to‐face intervention + printed materials Follow‐up 12 months post‐tx completion	E, DS	Usual care	Decisional regret Option regret Outcome regret	NS NS NS	NR NR NR	Decision aid did not significantly improve outcomes when compared with usual care	88% recruitment rate Compliance 100% 94% intervention and 91% controls had follow‐up at end of intervention
Victorson 2016 USA	43 men with low‐risk localised disease on active surveillance M age 69‐71 years	1. Mindfulness‐based stress reduction training Delivered by trained and experienced mindfulness instructor 8 weekly group face‐to‐face sessions Follow‐up 12 months post‐baseline	1. CB, R 2. E	2. Access to a book on mindfulness Self‐administered	PCa‐related anxiety Mental health	NS NS	NR NR	Mindfulness‐based stress reduction training did not significantly improve PCa anxiety or mental health when compared with access to a book on mindfulness	37% recruitment rate (refusal because of distance and lack of time) 88% of mindfulness intervention arm and 89% of mindfulness information arm had follow‐up at end of intervention
Weber 2004 USA	30 men ≤6 weeks since prostatectomy resulting in urinary and sexual dysfunction M age 58 years	Peer support Delivered by peer—a long term (> 3 years) PCa survivor who had undergone a prostatectomy that resulted in urinary and sexual dysfunction 8 individual face‐to‐face sessions over 8 weeks Follow‐up 8 weeks post‐baseline	PS	Usual care	Depression Self‐efficacy Sexual function Sexual bother	NS NS NS *P* = 0.014	NR NR NR NR	Peer support significantly reduced sexual bother when compared with usual care	49% recruitment rate (42% non‐responders and 9% refused) 12% withdrew from intervention Attendance rate 100% for intervention Qualitative assessment only of intervention acceptability
Weber 2007 a, b USA	72 men ≤3 months since dx and 6 weeks since prostatectomy Stage T1‐2 M age 60 years	Peer support Delivered by peer with long term PCa survivor who had undergone a prostatectomy at least 3 years prior to the study and had experienced similar tx side effects as the participants 8 Individual face‐to‐face sessions over 8 weeks Follow‐up 8 weeks post‐baseline	PS	Usual care provided by their urologist	Depression Self‐efficacy Mental health	*P* = 0.03 *P* = 0.005 *P* = 0.006#	NR NR NR	The peer support intervention significantly reduced depression and increased self‐efficacy regarding adjusting after PCa when compared with usual care # result excluded because of odds ratios of 0.0 which indicated results were problematic	53% recruitment rate (33% refused or did not respond, 14% excluded because of geographic location) Maximum 2 men withdrew from study as relocated – unclear from which group 88% mean attendance
Wootten 2015, 2016 Australia	142 men <5 years since tx 88% radical prostatectomy M age 61 years	1. Online psycho‐educational intervention (*PsychE*) Self‐administered 6 individual sessions over 10 weeks 2. Online psycho‐educational intervention + access to moderated peer online forum (*PsychE + F*) Self‐administered 6 individual sessions over 10 weeks Follow‐up 10 weeks (end of intervention)	1. E, CB, C 2. E, CB, PS, C 3. PS	3. Moderated peer online forum access (*F*) Self‐administered Individually accessed over 10 weeks	Distress *PsychE vs PsychE + F* *PsychE vs F* *PsychE + F vs F* PCa‐related worry Decisional regret *PsychE vs PsychE + F* *PsychE vs F* *PsychE + F v F* Erectile function Masculine self‐esteem Sexual satisfaction *PsychE v PsychE + F* *PsychE v F* *PsychE + F v F* Follow‐up 6 months post‐baseline	*P* = 0.02 NS NS *P* = 0.02 NS *P* = 0.05 NS NS *P* = 0.046 NS NS *P* = 0.028 NS NS NR	*η* ^2^ = 0.07 NR NR NR *η* ^2^ = 0.06 NR NR NR NR NR NR *η* ^2^ = 0.045 NR NR Difference 1.24 95% CI (0.25‐2.22)	The combined online psycho‐educational intervention + moderated peer forum significantly reduced distress and decision regret, and significantly improved sexual satisfaction when compared with moderated peer forum alone	30% withdrew from *PsychE* arm, 27% withdrew from *PsychE + F* arm and 23% withdrew from *F* only arm Mean 60% of psycho‐educational content completed in *PsychE* arm and mean 57% completed in *PsychE + F* arm Mean 1‐2 forum posts/user for *PsychE + F* intervention Mean 2‐3 forum posts/user for *F* only intervention
Yanez 2015 USA	74 men with advanced disease at dx who received ADT in last 6 months M age 69 years	1. CB stress management + relaxation/stress reduction techniques Delivered by ≥ masters level therapist 10 weekly group online sessions Follow‐up 6 months post‐baseline	1. E, CB, R, PS, C 2. E	2. Health promotion attention‐control (HP) Delivered by ≥ masters level therapist 10 weekly group online sessions	Depression Cancer‐related distress Cancer‐related QoL	NS NS NS	Cohen's d 0.5 0.2 0.3	The 10‐week CB stress management intervention lowered depression levels with a moderate effect size when compared with health promotion control	31% recruitment rate (refusal because of: time involved or lack of interest) 66% attendance for CB stress management and 82% for HP intervention (*P* = 0.04) Mean acceptability scores for both interventions were between liking the study “quite a bit” and “a lot “
Zhang 2006, 2007 USA	29 men ≥6 months (M 19‐22 months) since prostatectomy with post‐prostatectomy urinary incontinence Stage I–III M age 61‐62 years	Social support group + pelvic floor muscle exercises with biofeedback Delivered by a licenced health psychologist 6 bi‐weekly group face‐to‐face over 3 months Follow‐up 3 months post‐baseline	E, PS, C	Pelvic floor muscle exercises with biofeedback	Symptom distress Illness intrusiveness Mood	NS NS NS	NR NR NR	Addition of the social support group did not improve outcomes	57% recruitment rate (3 withdrew because of work schedules) 100% intervention and 87% controls had follow‐up at end of intervention

#Treatment is reported if ≥80% of men received it, with the exception of ADT where the percentage of men currently receiving ADT was reported. *Precision of effect and size of effect correspond to the longest reported follow‐up. ADT, Androgen deprivation therapy; C, Communication; CB, Cognitive‐behavioural; DS, Decision Support; Dx, Diagnosis; E, Education; EBRT, External beam radiation therapy; ED, Erectile dysfunction; M, Mean; NR, Not reported; NS, Not significant; PCa, Prostate cancer; PS, Peer Support; QoL, Quality of Life; R, Relaxation; SC, Supportive Counselling; Tx, treatment.

**Table A2:** Trials comprising couple‐focused interventions (*N* = 14)

Study	Couples#	Intervention	Intervention components	Comparator	Relevant outcomes	Precision of effect *	Size of effect *	Key findings	Acceptability
Campbell 2007 USA	30 African American couples (83% married) Men <4 years since tx (~93% prostatectomy) or start of watchful waiting M age years: 62 (patient) and 59 (partner)	Partner assisted coping skills training for survivors and their partners Delivered by African American doctoral level medical psychologists 6 ~weekly dyadic telephone sessions Follow‐up ~6 weeks post‐baseline (end of intervention)	E, CB, R, C	Usual care	*Patients* Mental health Sexual QoL Sexual function Sexual bother Self‐efficacy	NS NS NS NS NS	Cohen's d 0.0 0.3 0.3 0.5 0.2	For patients, coping skills training improved sexual bother with moderate effect size when compared with usual care	25% recruitment rate 75% of dyads completed intervention 60% intervention and 90% control had follow‐up at end of intervention Qualitative assessment only of intervention acceptability
					*Partner* Caregiver strain Mood Anger Confusion Depression Fatigue Anxiety Vigour Self‐efficacy	NS NS NS NS NS NS NS NS	Cohen's d 0.3 0.0 0.3 0.5 0.4 0.3 0.4 0.1	For partners, coping skills intervention improved depressed mood with a moderate effect size when compared with usual care	
Canada 2005 USA	51 couples (100% female; married/living together) Men ≤60 months since tx with ED 57% surgery; 31% radiation therapy Stage A‐C M age years: 65‐66 (patient) and 61‐62 (partner)	1. Sexual counselling—couple Delivered by psychologist or counsellor 4 dyad face‐to‐face sessions Follow‐up 6 months post‐intervention	1. E, CB, C 2. E, CB, C	2. Sexual counselling—patient only Delivered by psychologist or counsellor 4 individual face‐to‐face sessions	*Patients* Distress Sexual QoL Marital satisfaction Utilisation of tx for ED	NS NS NS NR	NR NR NR NR	Couples sexual counselling did not significantly improve patient outcomes when compared with patient only sexual counselling	66% completed couple intervention; 57% completed patient only intervention 21% withdrew because of high marital distress, 9% discomfort with explicit sexual topics, 6% scheduling conflicts 61% attended all 4 sessions
					*Partners* Distress Sexual function Marital satisfaction	NS NS NS	NR NR NR	Couples sexual counselling did not significantly improve partner outcomes when compared with patient only sexual counselling	
Chambers 2015 Australia	189 couples (100% female partners) Men with localised disease prior to (74%) or ≤12 months since prostatectomy M age years: 63 (patient) and 60 (partner)	1. Peer‐delivered telephone support Delivered by PCa survivors Recruited pre‐surgery: 8 dyadic (with partner) telephone sessions: 2 pre‐surgery +6 post‐surgery over 22 weeks Recruited post‐surgery: 6 dyadic (with partner) telephone sessions over 22 weeks 2. Nurse‐delivered telephone counselling Delivered by PCa nurse counsellors Recruited pre‐surgery: 8 dyadic (with partner) telephone sessions: 2 pre‐surgery +6 post‐surgery over 22 weeks Recruited post‐surgery: 6 dyadic (with partner) telephone sessions over 22 weeks Follow‐up 12 months post‐recruitment	1. E, CB, PS, C 2. E, CB, SC, C, DS	Usual care	*Patients* Sexual function Sexual supportive care needs Sexual self‐confidence Masculine self‐esteem Marital satisfaction Intimacy Use of ED tx	NS NS NS NS NS NS *P* < 0.01	NR NR NR NR NR NR NR	Patients in the peer intervention were 3.14 times more likely to use ED tx when compared with usual care (*z* = 2.41, *P* = 0.016) Patients in the nurse‐led intervention were 3.67 times more likely to use ED tx when compared with usual care (*z* = 2.64, *P* = 0.008)	47% recruitment rate At 6‐months post‐recruitment 8% peer‐delivered arm, 5% nurse‐delivered arm and 6% controls withdrew because no longer interested 88% (8 sessions) or 100% (6 sessions) median attendance for both peer‐ and nurse‐delivered interventions High helpfulness ratings for all interventions (1 not at all to 10 extremely) (Nurse intervention: Patient M 8.67, Partner M 8.33; Peer intervention: Patient M 7.74, Partner M 7.47)
					*Partner* Sexual function Sexual supportive care needs Marital satisfaction Intimacy	NS NS NS NS	NR NR NR NR	Peer or nurse‐delivered interventions did not significantly improve outcomes when compared with usual care	
Couper 2015 Australia	62 couples (100% female spouses) Men ≤12 months post‐dx Stage T1‐3 (19% T3) Median age years: 65 (patient) and 61 (partner)	Cognitive existential couple therapy Delivered by mental health professionals 6 weekly dyadic face‐to‐face sessions Follow‐up 9 months post‐baseline	CB, SC	Usual care	*Patients* Cancer‐related distress Distress Well‐being Relationship function	NS NS NS NS	NR NR NR NR	Cognitive existential couple therapy did not significantly improve outcomes for patients	18% consented to assessment for eligibility 7% dyads withdrew because of unacceptability of programme 100% median attendance rate
					*Partner* Cancer‐related distress Distress Well‐being Relationship function	NS NS NS *P* = 0.009	NR NR NR *η* ^2^ = 0.25	For partners, cognitive‐existential couple therapy significantly improved relationship function when compared with usual care	
Giesler 2005 USA *Reported patient data only*	99 couples (96% female spouses) Men ≤2 weeks post‐tx Stage T1a‐T2c Patient M age 64 years	Post‐tx nursing support Delivered by oncology nurse 6 monthly dyadic (with partner) sessions; 2 face‐to‐face and 4 telephone sessions Follow‐up 12 months post‐tx	E, C	Standard care	Mental health Sexual function Sexual limitation Sexual bother Depression Cancer worry Dyadic satisfaction Dyadic cohesion	NS NS *P* = 0.02 NS NS *P* = 0.03 NS NS	Effect size ‐0.1 0.4 0.5 0.2 0.2 0.5 0.4 0.1	For patients, post‐tx nursing support significantly reduced with a moderate effect size cancer worry and the extent to which sexual dysfunction interfered with spousal role activities when compared with standard care	48% recruitment rate Attrition rates reportedly similar in both groups
Lambert 2016 Australia	42 couples (97% married/ defacto) Men with early‐stage disease ≤4 months since dx, and patient or partner had distress thermometer score ≥ 4 M age years: 63‐64 (patient) 59‐60 (partner)	Coping skills for couples and relaxation Self‐administered Dyadic booklet, CD and DVD + 2 months use of the materials +4 telephone calls from a research assistant over 2 months to review and monitor use of materials Follow‐up 2 months post‐baseline (end of intervention)	E, R, C	Minimal ethical care Printed materials + 4 telephone calls over 2 months to review and monitor use of materials	*Patient* Anxiety Depression Self‐efficacy Mental health Cancer‐specific distress Uncertainty Relationship satisfaction Illness appraisal	NS NS NS NS NS NS NS NS	Difference −0.28 0.71 −4.41 −0.05 NR 4.60 NR NR	Coping skills and relaxation intervention did not significantly improve patient outcomes when compared with minimal ethical care	37% recruitment rate; 42% refused or did not respond (24% not interested, 7% too busy) No withdrawals during intervention in intervention arm 100% attendance rate for 91% (maximum) intervention arm and 74% (maximum) control arm
					*Partner* Anxiety Depression Self‐efficacy Mental health Caregiver QoL Cancer‐specific distress Uncertainty Relationship satisfaction Illness appraisal Threat Challenge Harm/loss Benign	NS NS NS NS NS NS NS NS NS *P* < 0.05 NS NS	Difference 0.62 1.17 2.17 −0.04 NR NR −3.51 NR −1.13 2.94 0.26 1.05	Partners who received coping skills intervention had significantly worse challenge appraisal scores than partners who received minimal ethical care	
Manne 2011 USA	71 couples (97% female; 97% spouses) Men ≤12 months since dx Stage 1‐2 M age years: 60 (patient) and 56 (partner)	Intimacy‐Enhancing Therapy (IET) Delivered by therapists 5 dyadic (with partner) face‐to‐face sessions over 8 weeks Follow‐up 8 weeks post‐baseline (end of intervention)	E, CB, SC, C	Usual care	*Patients* Distress Well‐being Cancer‐specific distress Cancer concerns Relationship satisfaction Intimacy	NS NS NS NS NS NS	NR NR NR NR NR NR	For a subgroup of patients with higher baseline cancer concerns, the IET intervention was predicted to significantly improve cancer concern when compared with usual care (*P* = 0.02)	21% recruitment rate (did not participate because of time required, or believed would not benefit) 22% did not attend any sessions (unclear if withdrew or not) 73% attendance ≥80% of sessions Intervention success M 3.2 (3 quite successful, 4 extremely successful) Intervention helpfulness M 4.2 (5 strongly agree)
					*Partners* Distress Well‐being Cancer‐specific distress Cancer concerns Relationship satisfaction Intimacy	NS NS NS NS NS NS	NR NR NR NR NR NR	For a subgroup of partners with higher baseline cancer‐specific distress, the IET intervention was predicted to significantly improve cancer‐related distress compared with usual care (*P* = 0.02) For a subgroup of partners with lower baseline relationship satisfaction (*P* = 0.002) and intimacy (*P* = 0.001), the intervention was predicted to significantly improve these outcomes compared with usual care For a subgroup of partners who had higher baseline levels of relationship satisfaction (*P* = 0.04) and intimacy (*P* = 0.02), the intervention was predicted to significantly reduce these outcomes compared with usual care	
McCorkle 2007 USA	107 couples (100% female spouses) Men immediately prior to radical prostatectomy 30% depressive symptoms (patient); 25% (partner)	Post‐tx nursing support for patient/partner dyad during an 8‐week period immediately following hospital discharge after radical prostatectomy Delivered by advanced practice nurse 8 weekly dyadic face‐to‐face sessions and 8 weekly telephone calls (16 contacts over 8 weeks) Follow‐up 6 months post‐surgery	E, C	Usual care	*Patients* Depression Sexual function	NS NS	NR NR	Post‐tx nursing support did not significantly improve patient outcomes when compared with usual care	7% of eligible dyads withdrew pre‐randomisation
					*Partners* Depression Relationship function Sexual function	NS NS *P* = 0.048	NR NR NR	Partners receiving post‐tx nursing support had significantly higher distress related to sexual function when compared with usual care (however, baseline sexual function not assessed)	
Northouse 2007 USA	235 couples (100% female spouses) Men ≤2 months since dx and 60% new dx; 14% detection of biochemical recurrence; 21% metastatic disease M age years: 63 (patient) and 59 (partner)	Supportive education for couples Targeted at disease phase and tailored to the needs of each couple Delivered by masters‐prepared nurses 5 bi‐weekly dyadic sessions: face‐to‐face (3) and telephone call (2) Follow‐up 12 months post‐intervention	E, R, C	Standard care	*Patients* Mental health Cancer‐related QoL Illness appraisal Uncertainty Hopelessness Self‐efficacy Symptom distress Sexual QoL	NS NS NS NS NS NS NS NS	Effect size −0.1 0.0 0.0 0.0 0.0 −0.1 0.1 0.0	Supportive education did not significantly improve patient outcomes or result in a moderate or large effect size when compared with standard care	69% recruitment rate (7% refused intervention assignment; 5% did not complete intervention 1% refused control assignment) 87% intervention and 92% control had follow‐up at end of intervention
					*Partners* Mental health Cancer‐related QoL Uncertainty Hopelessness Self‐efficacy Symptom distress Partner's sexual symptoms causing problems	NS NS NS NS *P* = 0.02# NS NS	Effect size −0.1 0.1 −0.1 −0.2 0.3 −0.1 −0.0	Supportive education intervention did not significantly improve partner outcomes or result in a moderate or large effect size when compared with standard care #Authors considered p < 0.01 as significant given multiple comparisons.	
Robertson 2016 UK	43 couples (98% female partners) Men dx 11 weeks to 4 years since surgery and with sexual dysfunction Patient M age ~64 years	Couple‐based relational psychosexual treatment Delivered by accredited counselling or psychotherapy practitioners 6 x 3‐4 weekly dyadic face‐to‐face sessions Follow‐up 6 months post‐intervention	E, CB, SC, C	Usual care Usual follow‐up hospital appointment	*Patient* and *Partner* Anxiety Depression Relationship function Patient Sexual bother	NR NR NR NR	NR NR NR NR	NR (no comparative results reported)	37% consented to assessment for eligibility; 38% of those eligible agreed to participate 24% withdrew from intervention and 23% withdrew from control 67% attended all 6 intervention sessions
Schover 2012 USA	100 couples (100% female partners; 97% spouses) Men 3 months—7 years since tx Stage T1‐T3 with erectile dysfunction (ED) Patient M age 64 years	1. Face‐to‐face sexual counselling Delivered by therapist 5 dyadic sessions (3 face‐to‐face, 2 telephone) over 12 weeks + printed handouts of materials on website 2. Internet‐based sexual counselling Delivered by therapist Dyadic self‐administered online materials with email contact and 2 telephone calls over 12 weeks Follow‐up 12 weeks post‐intervention	1. E, CB, SC, C, DS 2. E, CB, SC, C, DS	Waitlist control	*Patient* and *Partner* Distress Relationship satisfaction Sexual function and satisfaction	NR NR NR	NR NR NR	NR (no comparative results reported)	28% face‐to‐face and 13% internet‐based arm withdrew during intervention 75% face‐to‐face, 82% internet‐based and 90% controls followed‐up at end of intervention
Thornton 2004 USA	80 patients and 65 partners (100% female spouses) Men scheduled for prostatectomy Stage A‐C (17% Stage C) with baseline, 3 weeks post‐surgery and 1 year post‐surgery data M age years: 61 (patient) and 57 (partner)	Pre‐surgical communication enhancement Delivered by trained counsellor 1 dyadic (with partner) face‐to‐face session Follow‐up 1 year post‐surgery	SC, C	Standard care Basic information about surgery Delivered by a nurse	*Patients* Mental health PCa‐related QoL Sexual function Positive affect Negative affect Cancer‐specific stress Stress Relationship satisfaction	NS NS NS NS NS NS NS NS	NR NR NR NR NR NR NR NR	Pre‐surgical communication enhancement intervention did not significantly improve patient outcomes when compared with standard care	51% recruitment rate (47% did not participate because they were too busy) Compliance 100%
					*Partners* Mental health Positive affect Negative affect Cancer‐specific stress Stress Relationship satisfaction	NS NS NS NS NS NS	NR NR NR NR partial *η* ^2^ = 0.12 NR	For partners, the communication enhancement intervention reduced stress with a moderate effect size when compared with standard care	
Titta 2006 Italy *Reported patient data only*	57 patients and partners (100% female) Men 20‐41 days since prostatectomy (88%) Stage I‐II or cystectomy (8%) who requested sexual rehabilitation and responsive to and trained to administer PGE1‐intracavernous injections Patient M age 63.5 years	Intracavernous injection‐focused sexual counselling for couples following patient training in PGE1‐intracavernous injections Deliverer of sexual counselling NR Six 3‐monthly dyadic face‐to‐face sessions Follow‐up 18 months post‐surgery	E, SC, C	Control Partner invited to follow‐up visits every 3 months	Sexual function Erectile function Sexual satisfaction Orgasmic function Sexual desire	NS *P* < 0.05 *P* < 0.05 NS *P* < 0.05	NR NR NR NR NR	For patients, the intra‐cavernous injection‐focused sexual counselling intervention significantly improved erectile function, sexual satisfaction and sexual desire	100% intervention and 71% controls completed study 100% intervention and 71% controls had follow‐up at end of intervention
Walker 2013 Canada	27 couples (100% female married/defacto) Men starting ADT M age 73 years	Educational intervention for couples to maintain intimacy Delivered by researcher familiar with ADT 1 dyadic face‐to‐face session + booklet Follow‐up 6 months post‐enrolment	E	Usual care	*Patients* Intimacy Dyadic adjustment	NS NS	Cohen's d 0.6 1.0	For patients, educational intervention improved intimacy and dyadic adjustment with moderate and large effect sizes when compared with usual care	30% recruitment rate at main centre (did not participate because of being too busy or not interested) 100% compliance—men in intervention arm read at least part of booklet – all but 2 men read all of booklet
					*Partners* Intimacy Dyadic adjustment	NS NS	Cohen's d 0.0 0.5	For partners, educational intervention improved dyadic adjustment with a moderate effect size when compared with usual care (however, baseline levels of partner dyadic adjustment differed between arms and was not controlled for in analyses)	

#Treatment is reported if ≥80% of men received it, with the exception of ADT where the percentage of men currently receiving ADT was reported. *Precision of effect and size of effect correspond to the longest reported follow‐up. ADT, Androgen deprivation therapy; C, Communication; CB, Cognitive‐behavioural; DS, Decision Support; Dx, Diagnosis; E, Education; EBRT, External beam radiation therapy; ED, Erectile dysfunction; M, mean; NR, Not reported; NS, Not significant; PCa, Prostate cancer; PS, Peer Support; QoL, Quality of Life; R, Relaxation; SC, Supportive Counselling; Tx, Treatment.
